# Photonic sensing of organic solvents through geometric study of dynamic reflection spectrum

**DOI:** 10.1038/ncomms8510

**Published:** 2015-06-17

**Authors:** Yuqi Zhang, Qianqian Fu, Jianping Ge

**Affiliations:** 1 Shanghai Key Laboratory of Green Chemistry and Chemical Processes, School of Chemistry and Molecular Engineering, East China Normal University, Shanghai 200062, China; 2 Department of Chemistry, Tongji University, Shanghai 200092, China

## Abstract

Traditional photonic sensing based on the change of balanced reflection of photonic structures can hardly distinguish chemical species with similar refractive indices. Here a sensing method based on the dynamic reflection spectra (DRS) of photonic crystal gel has been developed to distinguish even homologues, isomers and solvents with similar structures and physical properties. There are inherent relationships between solvent properties, diffusion behaviour and evolution of reflection signals, so that the geometric characteristics of DRS pattern including ascending/descending, colour changes, splitting/merging and curvature of reflection band can be utilized to recognize different organic solvents. With adequate solvents being tested, a database of DRS patterns can be established, which provide a standard to identify an unknown solvent.

## Abstract (web summary)

Photonic sensing is a method for detecting individual chemical species, but can fail when they are sufficiently similar in physical properties. Here, the authors report a method that can distinguish even very closely related species, such as homologues and chemical isomers.

## Graphical abstract

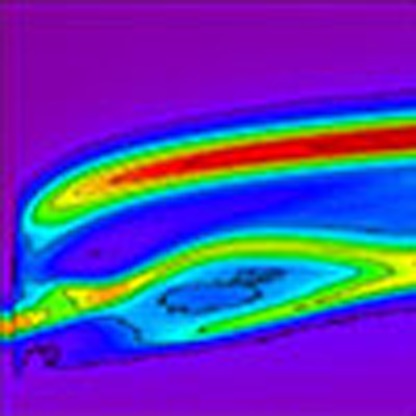

Responsive photonic crystals^1^,^2^,^3^,^4^ have attracted great interest due to their potential application in chemical sensing^5^,^6^,^7^,^8^,^9^,^10^,^11^,^12^,^13^,^14^, display units^15^,^16^, printing^17^,^18^,^19^, anticounterfeiting labels^20^,^21^,^22^, optical devices^23^,^24^,^25^, photo-catalysis^26^,^27^ and solar cells^28^,^29^. Among these applications, photonic crystal sensors have been widely investigated to detect ions^5^,^6^,^7^, molecules^8^,^9^, pH value^10^, solvent^11^, gas^12^,^13^, humidity^14^ and volatile organic compounds, which makes responsive photonic crystals an important member in the club of ‘smart materials'. Encouraged by the potential market of photonic sensors, various materials including artificial opal, inverse opal, Bragg mirror, polymeric and gel-like photonic composites are successively prepared^30^,^31^,^32^.

In the past years, photonic sensing for liquid and gaseous chemical species has been commonly realized by the shift of the balanced reflection peak and the change of structural colour compared with the original one. When the solvents infiltrate into the voids of opal solids, the effective refractive index of the whole colloidal crystal increases due to the replacement of air with solvent, which leads to the red shift of reflection accordingly. Both previous research^33^ and our experiments prove (Supplementary Fig. 1) that the final reflection wavelength (*λ*) has a linear relationship with the solvent's refractive index (*n*), so that the simulated straight line can be used to distinguish different solvents (Fig. 1a). This method is also applicable to the sensing of gaseous species as long as the opal or inverse opal solids are composed of porous materials^12^. However, the sensing based on the comparison of static reflection spectrum (SRS) in equilibrium state can hardly distinguish chemicals with similar or same refractive index, which suggests that the conventional method greatly limits the capability of colloidal crystals in fine sensing of chemical species.

In this work, we propose a new sensing method based on geometric study of the dynamic reflection spectrum (DRS) of photonic crystal gels. Typically, a hydrophilic photonic crystal gel composed of a SiO_2_ colloidal crystal array (CCA), poly(ethylene glycol) methacrylate (PEGMA) and ethylene glycol (EG) is used as the sensing material. Once the solvent contacts the gel, its reflection spectra are continuously recorded in the following 10 min, which are converted into a contour map with time (*t*) on the *x* axis, reflection wavelength (*λ*) on the *y* axis and reflection intensity (*R*) in colour for further study (Fig. 1b). Because the gel contains an inner solvent (EG) rather than air compared with opal solids, more physical properties of the analytes, such as polarity, viscosity, refractive index and even affinity to the PEGMA/EG gel, will affect its diffusion and polymer deformation, which leads to the infiltration of analyte along with lattice expansion or the extraction of inner solvent with lattice shrinkage. Therefore, the complex diffusion behaviour causes great diversity in DRS patterns. Compared with the traditional SRS method, abundant information can be obtained from DRS pattern, which can certainly help in tapping more the potential of photonic crystals in chemical sensing.

## Results

### Photonic crystal gel and diversified response to solvents

The photonic crystal gel for DRS sensing is prepared by fixing the metastable SiO_2_ CCAs dispersed in the mixture of PEGMA monomer and ethylene glycol through photo-polymerization^34^. The final volume ratios of SiO_2_ particles, EG and PEGMA are 40%, 38.5% and 21.5%, respectively. In the precursor, part of the SiO_2_ particles precipitate into colloidal crystals due to supersaturation, while the rest remain in Brownian motion in solution^35^. This microscopic phase separation makes the photonic gel finally a combination of crystalline SiO_2_ CCAs and amorphous stacking of SiO_2_ particles. After removing the EG content in gel, the phase separation structure can be verified by SEM images directly (Supplementary Figs 2 and 3). Twenty-two individual SEM images with an amplification of × 5,000 are spliced to show the whole cross section of a gel with thickness of 180 μm. One can clearly observe the ordered arrangement of SiO_2_ particles (highlighted by green), the random stacking of SiO_2_ particles and the boundary between the crystalline and amorphous zone. Since the amorphous zone is colourless and transparent like glass, the gel presents brilliant structure colours due to the high crystallinity of each crystalline domain, which provides a high-quality photonic material for chemical sensing.

**Figure 2:**
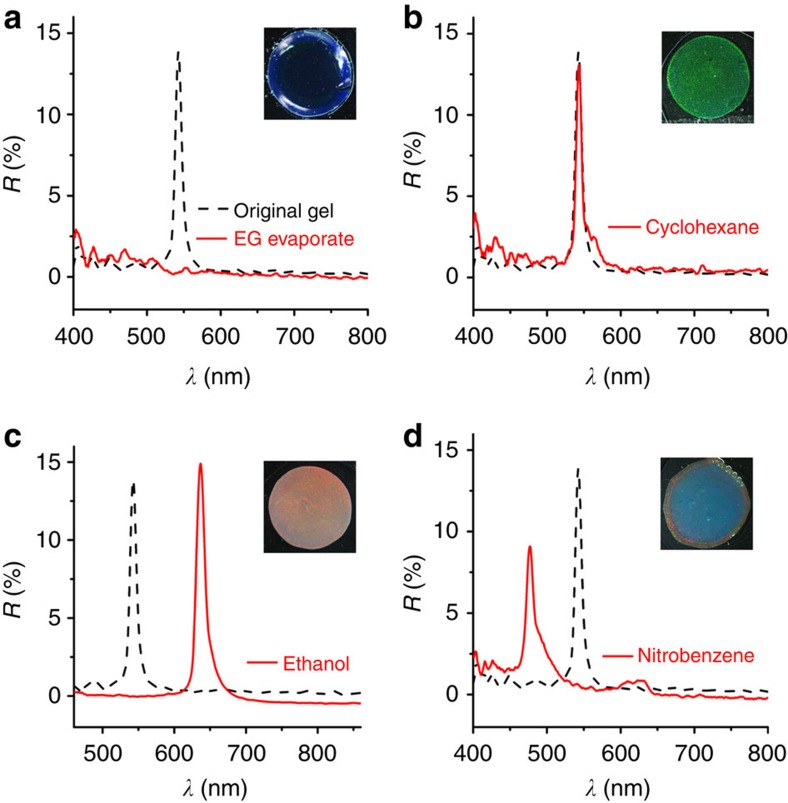
Typical optical response to different solvents. Reflection changes of a typical green photonic crystal gel (dash line) due to (**a**) EG evaporation and contact with (**b**) cyclohexane, (**c**) ethanol and (**d**) nitrobenzene.

The existence of inner solvent (EG) leads to complicated diffusion behaviour, which renders the gel a distinct response to different organic solvents (Fig. 2). The presence of inner solvent can be proved by thermal evaporation, through which the green gel turns into a transparent film and the reflection peak disappears accordingly due to the loss of EG and matching of *n* between SiO_2_ and PEGMA/EG matrix (*n*_SiO2_=1.46, *n*_PEGMA_=1.46 and *n*_EG_=1.43). When the gel is covered by a nonpolar analyte like cyclohexane, its reflection and structural colour remain unchanged as cyclohexane will not diffuse into the polar gel. It is not surprising that the gel turns red and redshifts its reflection when covered by a polar analyte like ethanol, because the lattice spacing expands due to the diffusion of ethanol and the swelling of photonic gel. However, it is unusual that the gel turns blue when it is covered by analytes such as nitrobenzene, probably because the lattice constant decreases due to the extraction of EG and thereby shrinkage of photonic gel.

**Figure 3:**
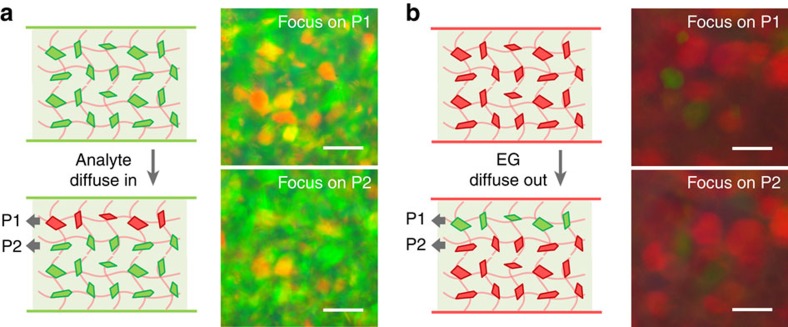
Determination of the direction of solvent diffusion. Microscopic images of colloidal crystals in the surface layer (P1) and inner layer (P2) of (**a**) a green and (**b**) a red photonic gel, when they are covered by (**a**) *i*-butanol and (**b**) acetyl acetate, respectively. Scale bars, 50 μm.

The judgements of solvent diffusion and extraction are supported by the spatial change of structural colours inside the gel. Since the gel is composed of transparent amorphous domains and colourful crystalline domains, one can directly observe the colloidal microcrystals in the surface layer (P1) or the inner layer (P2) by tuning the focus plane on a reflection-mode optical microscope. Generally, the crystals on focus have sharp edges while those under or over focus are quite blurry, which makes it possible to attribute each crystal to P1 or P2 plane. When a green gel is covered by butanol, the microcrystals in the surface layer turn red and those in the inner layer remain green, which demonstrates the diffusion of butanol into the gel from the surface layer (Fig. 3, Supplementary Fig. 4). For the case of acetyl acetate tested on a red gel, the surface crystals turn green while the inner crystals remain red, indicating the extraction of EG from the gel.

### Geometric characteristic of DRS pattern and solvent sensing

The use of gel-like sensing material diversifies the diffusion behaviour and leads to distinct DRS patterns, which provide multiple geometric criteria for the precise recognition of solvents. The collection of DRS pattern is described in Methods and Supplementary Fig. 5. Generally, DRS patterns will reveal the following geometric characteristics. (1) They show the shift of reflection wavelength (Δ*λ*) through a curved ‘reflection band', and the change of reflection intensity (Δ*R*) by the band colours. (2) The reflection band may split or merge due to the appearance of non-uniform distribution of solvent along the diffusion direction or the recovery of uniform distribution. Each reflection band actually indicates the reflection signals of colloidal crystals in the surface layer or inner layers, and the boundary of these layers keeps on moving until a balanced state is achieved. (3) The reflection band sometimes disappears owing to the matching of refractive index between silica colloids and the solvent-swelled PEGMA matrix. (4) The curvature of reflection band around the inflection point is related to the diffusion speed of solvent. The study of these geometric characteristics helps in understanding kinetic process of solvent diffusion and polymer swelling, and makes it possible to distinguish samples that cannot be recognized by the traditional SRS method.

**Figure 4:**
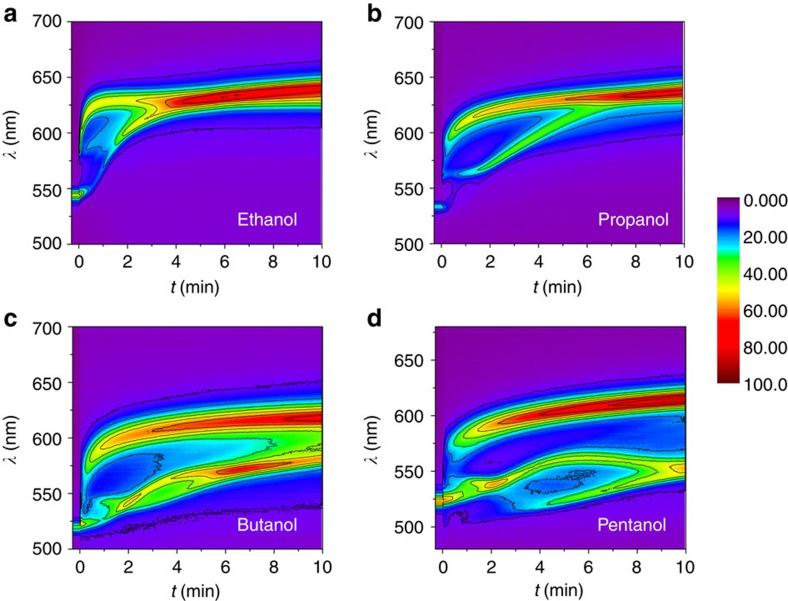
DRS patterns of alcohols. DRS patterns of homologues of alcohols including (**a**) ethanol, (**b**) propanol, (**c**) butanol and (**d**) pentanol.

The DRS method is capable of distinguishing homologues of alcohols according to their difference in polarity. It is known that ethanol, propanol, butanol and pentanol have nearly similar refractive indexes (Supplementary Table 1), which induce similar Δ*λ* in traditional SRS detection. However, their DRS patterns are quite different, so that one can easily attribute them to specific alcohols (Fig. 4). The common characteristics include two to three ascending reflection bands with increased *λ* and *R*, the splitting of initial reflection bands, and their merging within or possibly after 10 min. When the alcohols diffuse into the gel, the colloidal crystals in surface layer expand more and faster than those in the inner layer due to prior swelling. Therefore, the reflection splits into two higher bands above the original, which can be attributed to reflection of the surface and inner layer, respectively. Once the diffusion and swelling reach a balanced state, the reflection bands will merge and go flat afterwards. Generally, the stronger the polarity that the alcohol has, the faster the rise in reflection band because the PEGMA/EG gel is more diffusible and swellable by polar solvents. In short, a sharp rise in the reflection band and an early appearance of merging point in the DRS pattern indicate a short-chain *n*-alcohol.

In addition to polarity, some physical properties, including the solvent's viscosity, refractive index and its affinity to PEGMA/EG gel, also have great influence on the DRS patterns. For example, glycerol and DMSO are both polar solvents with a large dielectric constant (*ɛ*_Gly_=42.5; *ɛ*_DMSO_=48.9) and almost the same refractive index (*n*_Gly_=1.475; *n*_DMSO_=1.477), but their DRS patterns are completely different due to different viscosities (*η*_Gly_=1412, *η*_DMSO_=1.996 mPa s) (Supplementary Fig. 6). When DMSO comes in contact with the gel, it gradually diffuses into the gel, which causes the splitting of reflection band into two high bands at 0 min and merging of these two bands at 4 min. The DRS pattern is similar to that of alcohol, except that the surface band jumps to a high position and then decreases due to over-swelling of DMSO in the surface layer. For glycerol, it would not diffuse into the gel because it is too viscous to do so. Considering the ‘similarity-intermiscibility' theory, the inner solvent (EG) is extracted and the gel shrinks accordingly, so that the original reflection band splits into two lower bands. Here the lowest band and the gradually disappeared band correspond to the reflection of the surface and inner gel, respectively.

As another example, the DRS method can distinguish EG and DEG based on their small difference in refractive index (*n*_EG_=1.432; *n*_DEG_=1.448). It is known that the disappearance of reflection band is caused by matching of refractive index between silica particles and the solvent-swelled PEGMA matrix. Therefore, the time taken for the band to disappear together with the evolution of band colours is different for EG and DEG (Supplementary Fig. 7). Furthermore, the affinity of analyte to the PEGMA/EG gel also plays an important role in determining the shape of the DRS pattern. Acetic acid and ethyl acetate have similar dielectric constant (*ɛ*_AA_=6.15; *ɛ*_EA_=6.02), similar viscosity (*η*_AA_=1.314, *η*_DMSO_=0.449 mPa s) and equal refractive index (*n*_AA_=*n*_EA_=1.372), which should generate similar DRS pattern. However, the former pattern is similar to that of alcohol with merging of two high reflection bands splitting from the original band, while the latter is composed of one ascending band in the bottom and two crossed bands on the top (Supplementary Fig. 8), because acetic acid, compared with ethyl acetate, has better affinity to PEGMA/EG.

### Simulation of diffusion speed for sensing butanol isomers

Intrinsically, the DRS detection of organic solvents is based on the relationship between solvent properties, diffusion behaviour and geometric characteristics of the DRS pattern. As a simple example, the DRS pattern of acetophenone is simulated to explain its diffusion behaviour. The instant reflection wavelength of photonic gel (*λ*_*t*_) can be calculated using equation (1), where *λ*_0_ is the initial reflection wavelength, and *V*_A_, *V*_0_ (30 μl), *n*_A_ and *n*_0_ are the volume and refractive index of infiltrated acetophenone and original gel, respectively. This equation was deduced based on the Bragg's Law, the refractive index of the multiple-component system, and the fact that diffusion of acetophenone only causes the lattice expansion along the normal direction of the gel. At any specific time, the volume fraction of acetophenone [*ϕ*_*t*(A)_] inside the gel can be described by equation (2). On the basis of these two equations, the ‘*λ*_*t*_–*t*' curve plotted from DRS pattern can be transformed into a ‘*ϕ*_*t*(A)_–*t*' curve, which is further simulated with a Michaelis–Menten model to achieve equation (3). *V*_max_ and *K*_m_ are two constants, so that the volume fraction of infiltrated acetophenone is a function of time (*t*) only. Through derivation operation, the diffusion speed of acetophenone (indicated by d*ϕ*_*t*(A)_/d*t*) can finally be expressed by equation (4) (Supplementary Discussion, Supplementary Fig. 9). The simulation indicates that the DRS pattern actually reflects the solvent diffusion behaviour via the time evolution of reflection signals. In DRS pattern, a sharp rise of *λ* and a large curvature near the inflection point usually indicate a fast solvent diffusion and polymer swelling, while in the simulated formula a smaller *C*_1_ indicates a larger initial speed of solvent diffusion, and a large *C*_2_ shows a small change of diffusion speed along with the time:


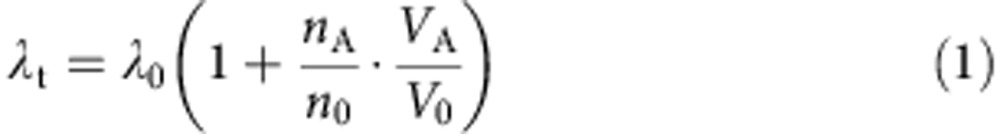



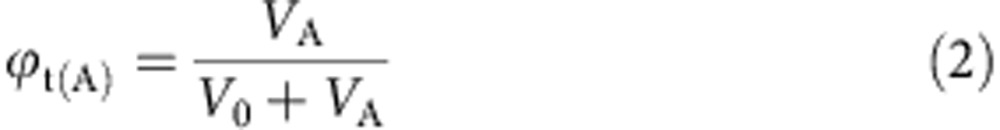



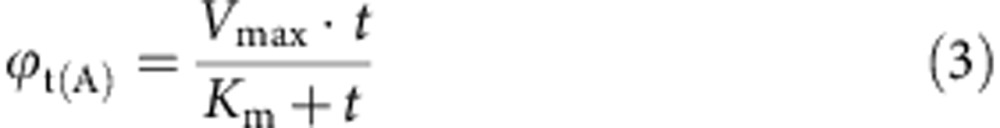



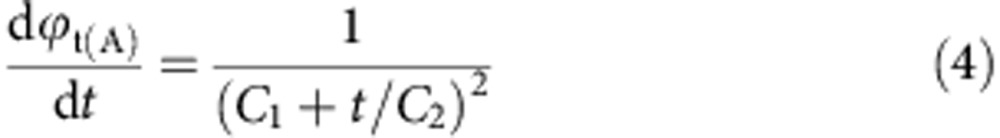


On the basis of these criteria, the DRS patterns have the potential to distinguish four isomers of butanols. *N*-, iso-, 2- and tert-butanol have very similar refractive index (1.399, 1.396, 1.397 and 1.384), similar viscosity (2.95, 4.0, 4.21 and 3.35) and comparable dielectric constant (17.1, 17.95, 15.5 and 11.4). Their DRS patterns are usually composed of two ascending reflection bands corresponding to the surface and inner layer, respectively (Supplementary Discussion, Supplementary Fig. 10). The simulations of ‘*ϕ*_*t*(A)_–*t*' curves are similar to the case of acetophenone, except that the solvent diffusion and lattice expansion in two layers need to be considered separately (Supplementary Fig. 11). In DRS pattern, it is easy to attribute the largest rise of inner reflection band to *n*-butanol, and the nearly flat inner band to *t*-butanol. Meanwhile, *i*-butanol and 2-butanol with similar inner band can be further distinguished by the curvature of surface band around the inflection point. On the basis of the simulation results, these four butanols can be distinguished by constant *C*_1_, which basically increase in the sequence of *n*-, iso-, 2- and tert-butanol (Supplementary Table 2).

### Category of DRS patterns and building database

**Figure 5:**
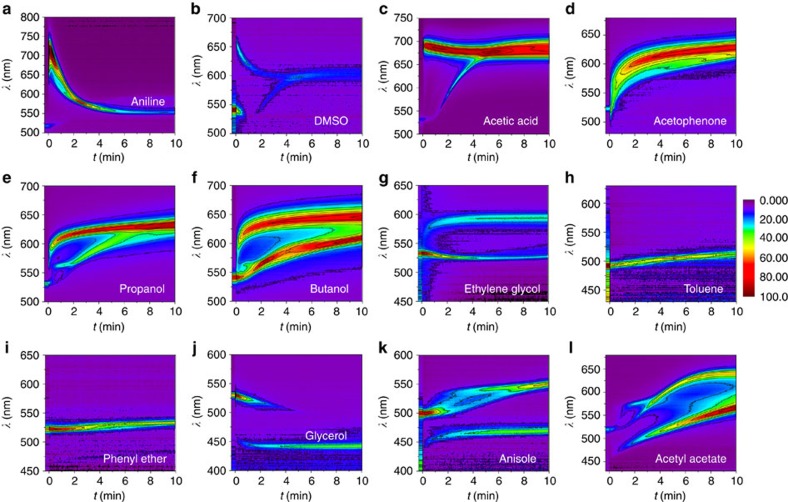
DRS patterns of various solvents. Twelve typical DRS patterns summarized from the gel's responses to various organic solvents.

In addition to the sensing of solvents with known formula, the DRS method could be used to determine an unknown solvent. In this work, we have investigated around 30 organic solvents including alkanes, alcohols, ethers, aldehydes, ketones, acid, esters, amine, nitriles and benzene derivatives. Twelve typical DRS patterns are observed (Fig. 5), which represent the optical response of photonic gel to (a) aniline, (b) dimethyl formamide (DMF)/dimethyl sulphoxide (DMSO), (c) methanol/acetic acid, (d) benzaldehyde/acetophenone, (e) ethanol/propanol, (f) butanol/pentanol, (g) ethylene glycol/diethylene glycol, (h) toluene/carbon tetrachloride, (i) phenyl ether/cyclohexane, (j) glycerol/dodecanol, (k) nitrobenzene/dichlorobenzene/anisole and (l) acetyle acetate/ethyl acetate/benzoyl chloride. For each solvent, we have used at least five separately prepared SiO_2_/PEGMA/EG photonic gels and recorded their response to the same solvent. One of the DRS patterns is listed in the main text, and three of them are shown in Supplementary Figs 12–22, named as #1, #2 and #3 to prove the repeatability. In the parallel measurement, all conditions including the preparation of photonic gel, the measurement of DRS pattern and the analyte solvent are kept the same, except that differently sized SiO_2_ particles may sometimes be used to fabricate the photonic gel. Although these photonic gels have different initial reflection wavelength, this has no effect on the recognition of a specific solvent because the criteria used to sense the solvent, that is, the geometric characteristics of the DRS pattern, are identical among all measurements.

**Figure 6:**
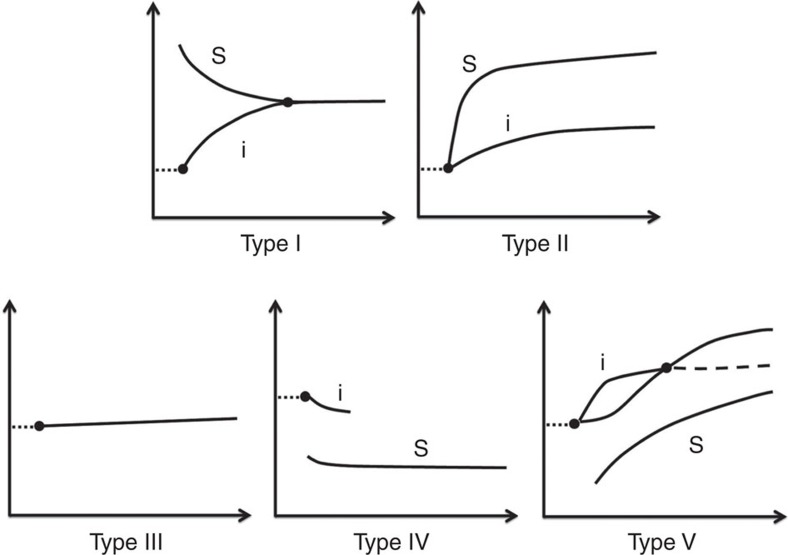
Five typical DRS patterns of a single-component solvent. Classification of DRS pattern according to the diffusion direction of tested solvents and inner solvent.

The DRS patterns can be categorized into five typical shapes (Fig. 6) according to the diffusion direction of the tested solvent and inner solvent EG (Table 1). The following discussion is based on the fact that the ascending or descending of the reflection band is majorly caused by the infiltration or extraction of solvent, because the change of refractive index cannot lead to a Δ*λ* around 100 nm. Generally, type I and II DRS patterns indicate that the tested solvents diffuse into the photonic gel, as proved by the rising of reflection bands. Type-I solvents (Fig. 5a–c) usually have large polarity or affinity to the PEGMA/EG gel, so that the surface photonic gel is overswelled by these solvents at the beginning, which gradually recovers the balanced state by squeezing out the excessive solvents to the inner layers. Type-II solvents (Fig. 5d–g) are general polar solvents with one or several ascending reflection bands due to sequential solvent infiltration. The original reflection band may split or not depending on which is fast between diffusion and polymer swelling. Generally, a sharp rising of reflection band and an early appearance of merging point indicate a solvent with larger polarity. Type-III solvents (Fig. 5h,i) are usually nonpolar organic solvents, so that they will not diffuse into the photonic gel and the EG inside the gel will not be extracted to them either. The typical DRS pattern is a flat reflection band or linear ascending band with very small slope. Type IV and V DRS patterns have a common feature that EG is extracted from the photonic gel, leading to a descending reflection band below the original one. Here the lower band corresponds to the reflection of surface layer. Type-IV solvents (Fig. 5j) could be viscous solvents, which cause the extraction of EG only, while the tested solvent scarcely diffuses in. However, type-V solvents (Fig. 5k,l) are usually less polar organic solvents, which gradually diffuse into the photonic gel along with the extraction of part of the EG from the gel, so that the DRS pattern actually shows a complicated solvent exchange process.

The DRS patterns of all tested organic solvents can be organized to build a database of standard patterns. By comparing the geometric characteristics with the standard pattern, one can determine an unknown solvent and obtain fundamental information about its polarity, viscosity and other characteristics. Combined with the traditional SRS method, photonic crystal sensing could be a more practically useful tool to realize precise recognition of organic species.

Certainly, the current sensing system still needs to be improved in many aspects such as the sensing speed and the recognition of solvent mixtures. Related research works are in progress and the preliminary results show that the sensing process can be shortened to 1 min when using a 40-μm photonic gel instead of the current 180-μm gel. With the decrease of gel thickness, the diffusion of solvent throughout the gel will require less time so that the sensing speed can be increased accordingly. For the recognition of solvent mixtures, the preliminary results suggest that the DRS pattern of the solvent mixture may retain or combine part of the geometric characteristics of their single component, which even generates new patterns outside the five categories. By studying the relationship between the DRS pattern of mixture and that of its component, it might be possible to determine the species and the volume ratio of an unknown solvent mixture directly from its DRS pattern (Supplementary Discussion, Supplementary Fig. 23).

## Discussion

A new photonic sensing method based on the geometric study of DRS has been developed to distinguish alcohol homologues, isomers and organic solvents with similar structures and physical properties. Since the sensing material is a photonic crystal gel composed of SiO_2_ particles, hydrophilic polymer (PEGMA) and solvent (EG), the analyte solvent may diffuse into the gel or extract the EG from the gel depending on its polarity, viscosity and even affinity to the gel. There are inherent relationships between solvent properties, diffusion behaviour and evolution of reflection signals, so that the geometric characteristics of DRS pattern including ascending/descending, colour changes, splitting/merging and curvature of reflection band can be utilized to recognize the analyte solvents. With adequate solvents being tested, a database of DRS patterns can be established, which provide standard patterns to identify an unknown solvent.

## Methods

The synthesis of SiO_2_/PEGMA/EG photonic crystal gel and its characterization are provided in Supplementary Methods. Generally, reflection spectra are continuously recorded in steps of 2 s by using the probe of an ultraviolet–visible spectrometer with incident and reflection angles fixed at 0°. The probe is faced upwards vertically in the holder, which is covered by a transparent glass slide. Meanwhile, the photonic crystal gel is fixed on another glass slide by an optical adhesive and placed on the first slide, so that the gel is actually sandwiched between two slides. After the reflection spectra are collected by the spectrometer, the tested solvent (200 μl) is added onto the surface of the photonic gel. The volume of solvent is adequate to soak the gel although part of the solvent will evaporate. After 10 min, about 300 reflection spectra are imported to the software to plot the DRS pattern.

## Additional information

**How to cite this article:** Zhang, Y. *et al.* Photonic sensing of organic solvents through geometric study of dynamic reflection spectrum. *Nat*. *Commun*. 6:7510 doi: 10.1038/ncomms8510 (2015).

## Supplementary Material

Supplementary Information Supplementary Figures 1-23, Supplementary Tables 1-2, Supplementary Discussion, Supplementary Methods and Supplementary References

## Figures and Tables

**Figure 1:**
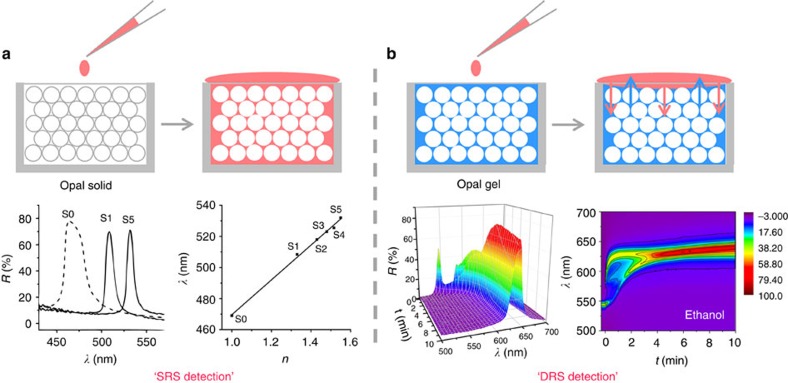
Comparison of photonic sensing using SRS or DRS strategies. (**a**) ‘Static reflection spectrum' detection of methanol (S1), ethylene glycol (S2), DMSO (S3), anisole (S4) and dichlorobenzene (S5) based on the redshift of balanced reflection wavelength compared with that of pure SiO_2_ opals (dash line, S0). (**b**) ‘Dynamic reflection spectrum' sensing of ethanol based on the time evolution of reflection of an opal gel presented in the form of a 3D surface map and contour map.

**Table 1:** Category of tested solvents according to the type of DRS patterns.

**DRS pattern**	**Solvent**
Type I	Aniline, DMF, DMSO, methanol, acetic acid
Type II	Benzaldehyde, acetophenone, ethanol–pentanol, ethylene glycol
Type III	Toluene, CCl_4_, phenyl ether, cyclohexane
Type IV	Glycerol, dodecanol
Type V	Nitrobenzene, dichlorobenzene, anisole, acetyl acetate, ethyl acetate, benzoyl chloride
