# Supplementary material for: Photonic sensing of organic solvents through geometric study of dynamic reflection spectrum
Source: Nat Commun. 2015 Jun 17;6:7510. doi: 10.1038/ncomms8510 (PMC4557364; doi:10.1038/ncomms8510)
Supplement: Supplementary Information — Supplementary Figures 1-23, Supplementary Tables 1-2, Supplementary Discussion, Supplementary Methods and Supplementary References [file ncomms8510-s1.pdf]

Supplementary Figures

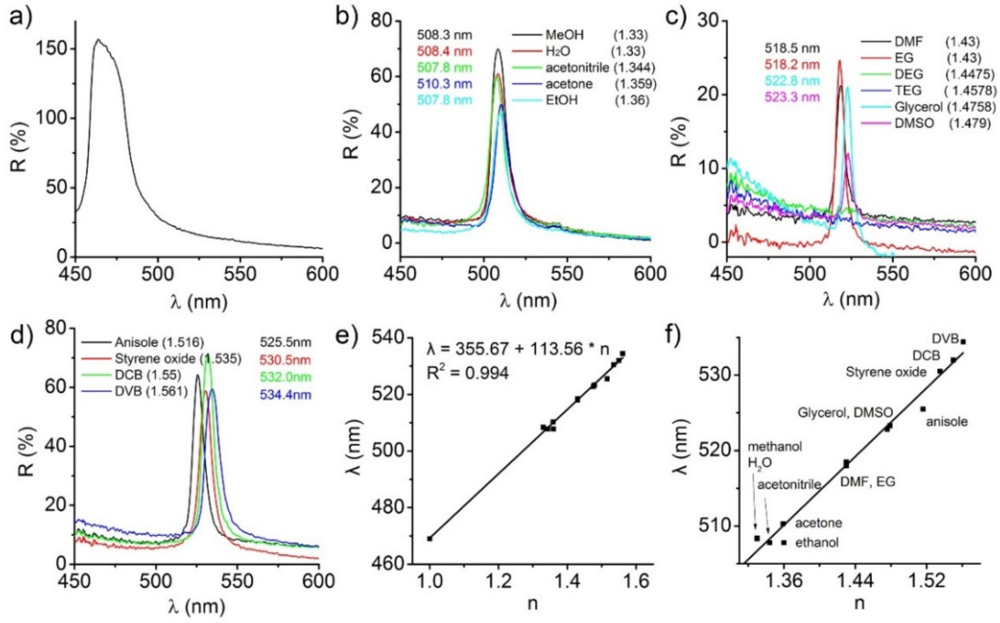

Supplementary Figure 1. Photonic sensing via SRS method. Reflection spectra of a) a dried SiO_2 opal and b-d) the SiO_2 opal infiltrated with different organic solvents, whose refractive indices change from 1.33 to 1.56. e-f) The linear relationship between λ and n can be used to distinguish solvent with different refractive index.

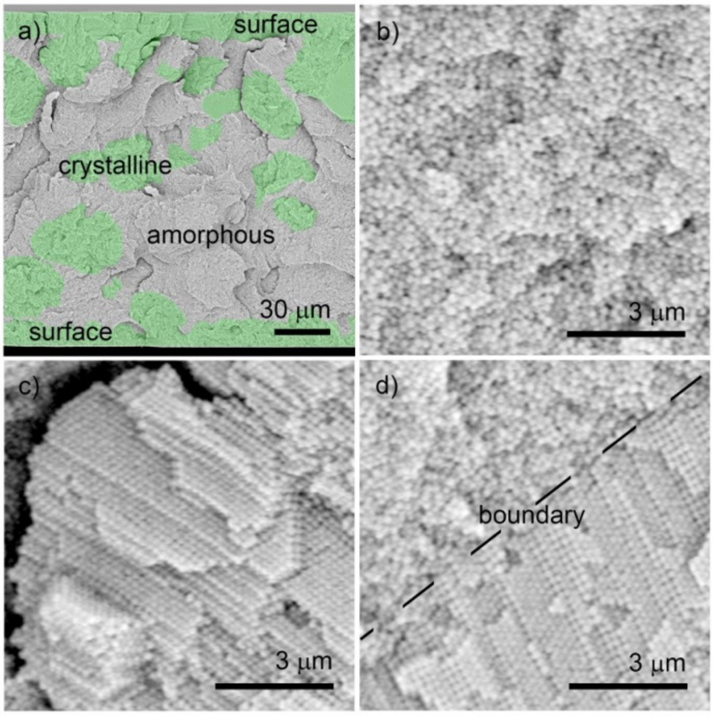

Supplementary Figure 2. SEM image of photonic crystal film. a) Cross-section SEM images of a dried SiO_2/PEGMA/EG colloidal crystal gel with crystalline zone highlighted in green and amplified SEM images of b) amorphous zone, c) crystalline zone and d) the boundary.

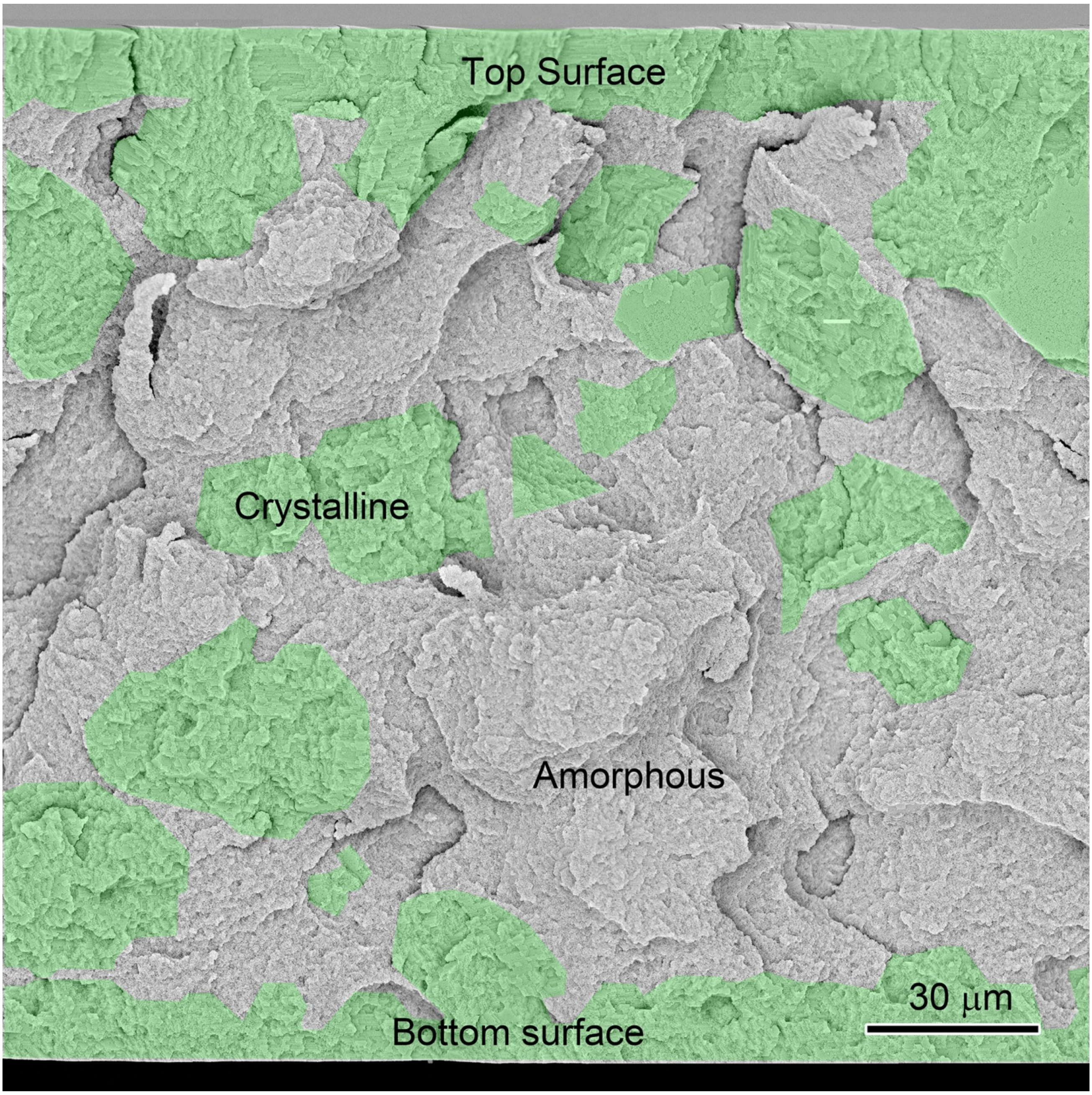

Supplementary Figure 3. High resolution SEM image of photonic crystal film. Amplified SEM image of the cross-section of the dried colloidal crystal film after removing the EG content. It is spliced by 22 individual SEM images with amplification of 5000×, which shows the SiO_2 colloidal crystals and amorphous stacking of SiO_2 particles from the top surface to the bottom surface.

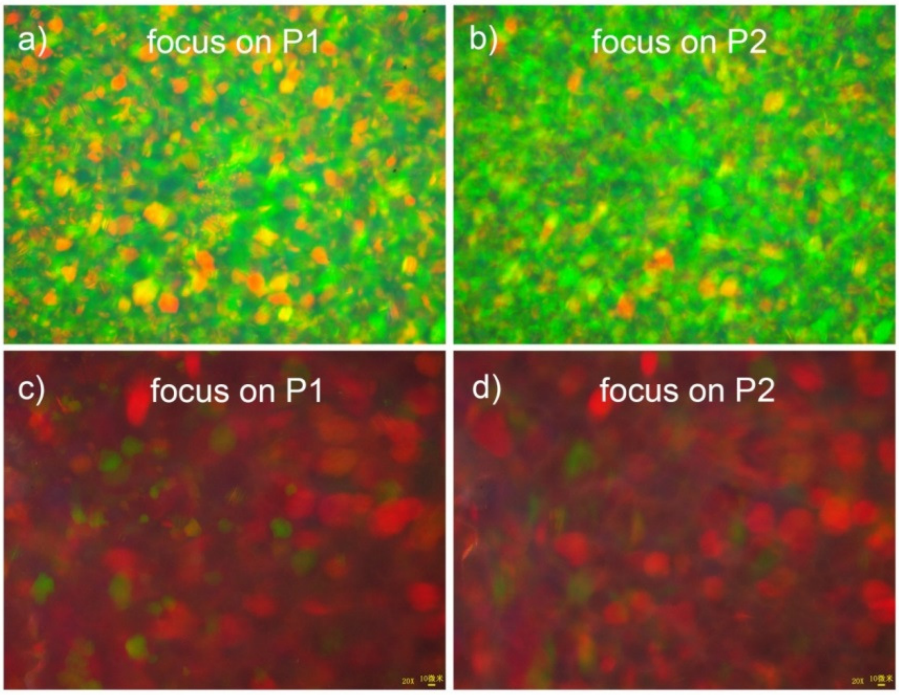

Supplementary Figure 4. Determination of solvent diffusion direction by optical microscope image. Optical microscope images of a) a green photonic gel infiltrated with iso-butanol and b) a red photonic gel infiltrated with acetyl acetate. The pictures are captured when the microscope is focus on the surface layer (P1) and inner layer (P2), respectively.

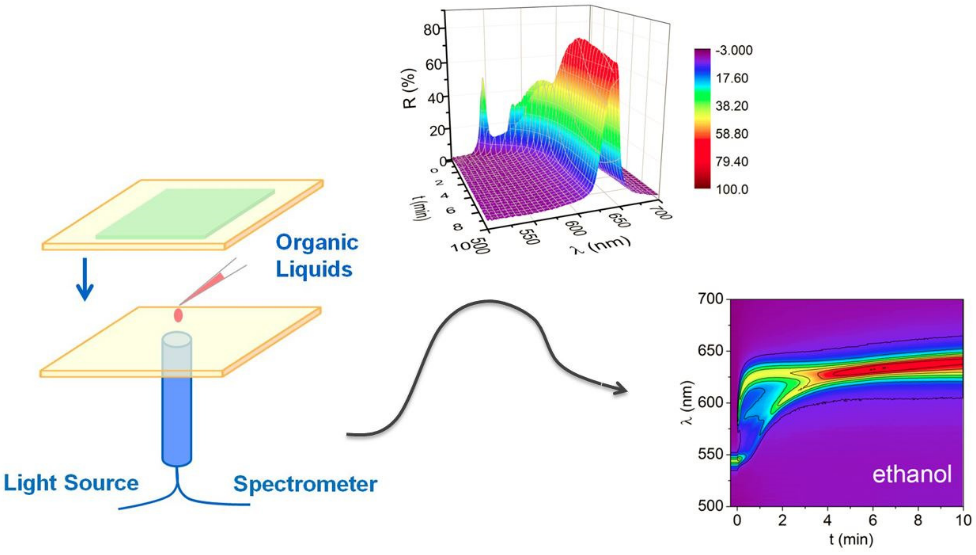

Supplementary Figure 5. Procedures of DRS photonic sensing. The experimental setup of light source, reflection probe, photonic gel and spectrometer. The dynamic reflection spectra are continuously recorded by the spectrometer, which are plotted to a 3D surface map and contour map with time as "*x*", reflection wavelength as "*y*", reflection intensity as "*z*" or characterized by colors.

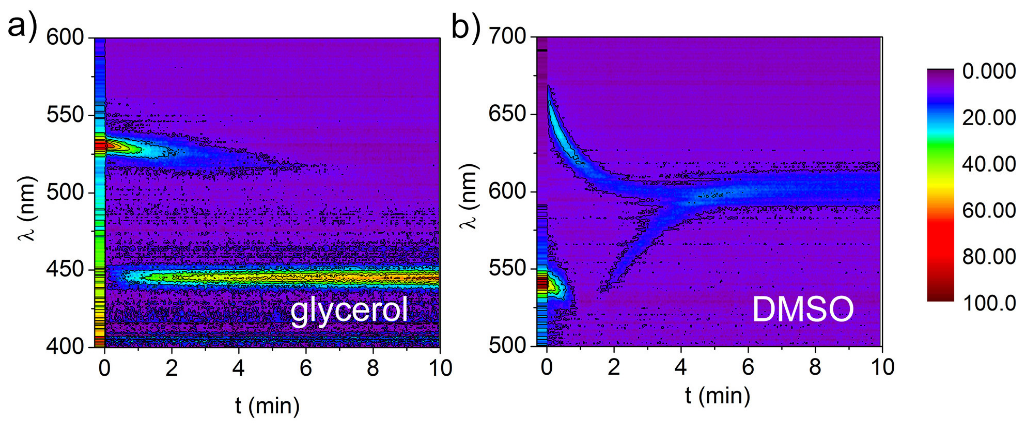

Supplementary Figure 6. DRS patterns of glycerol and DMSO. DRS pattern of a) glycerol and b) DMSO, which have close dielectric constant, almost same refractive index but different viscosity.

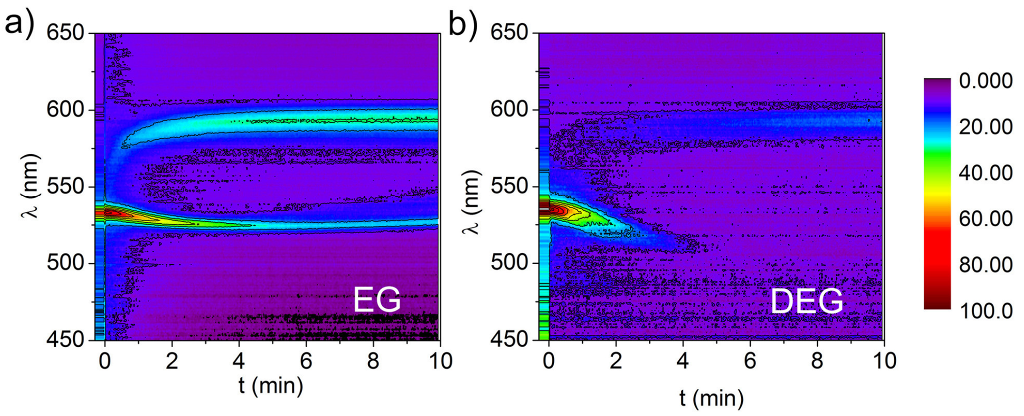

Supplementary Figure 7. DRS patterns of EG and DEG. DRS pattern of a) ethylene glycol and b) diethylene glycol, which have slightly different refractive index.

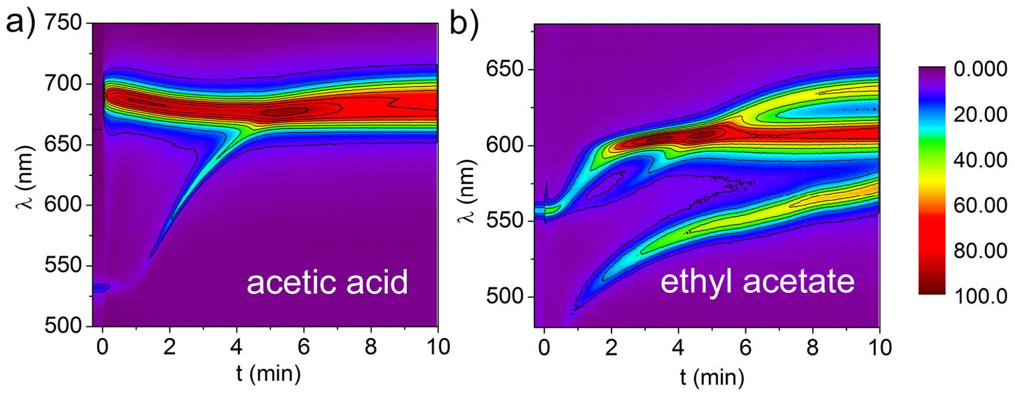

Supplementary Figure 8. DRS patterns of acetic acid and ethyl acetate. DRS pattern of a) acetic acid and b) ethyl acetate, which have close dielectric constant, similar viscosity, equal refractive index but different affinity to PEGMA/EG gel.

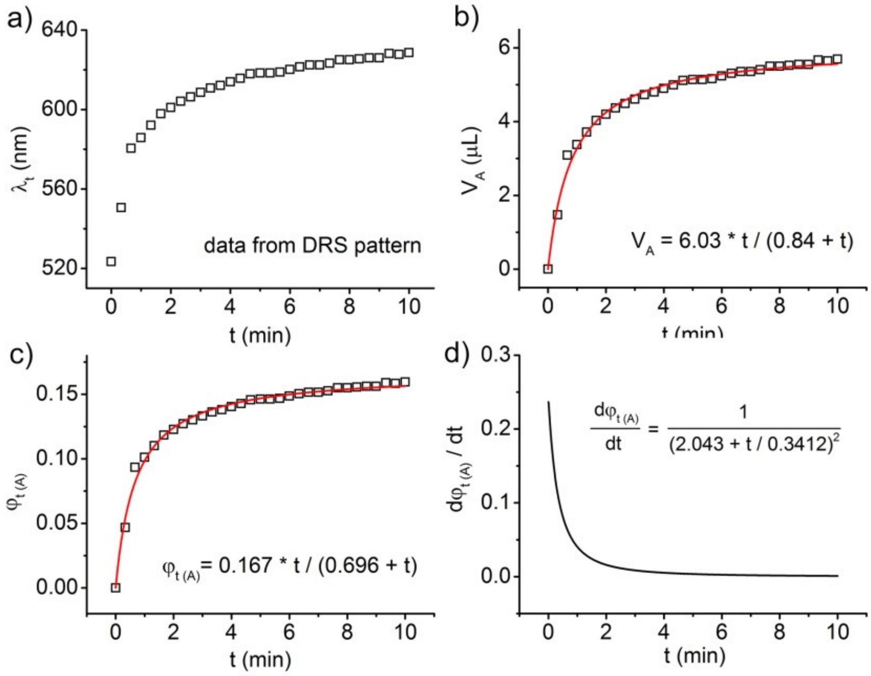

Supplementary Figure 9. Simulation of solvent diffusion speed from DRS pattern. Time evolution of a) reflection wavelength of photonic gel in response to acetophenone. Time evolution of b) volume, c) volume fraction and d) diffusion speed of infiltrated acetophenone and corresponding simulations.

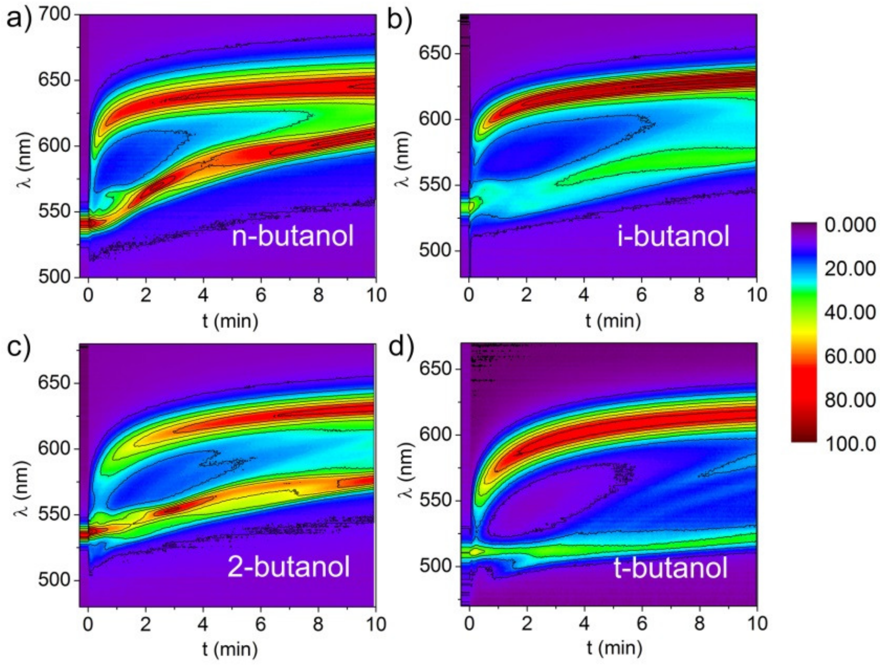

Supplementary Figure 10. DRS patterns of butanol isomers. DRS patterns of isomers of butanols including a) n-butanol, b) i-butanol, c) 2-butanol and d) t-butanol.

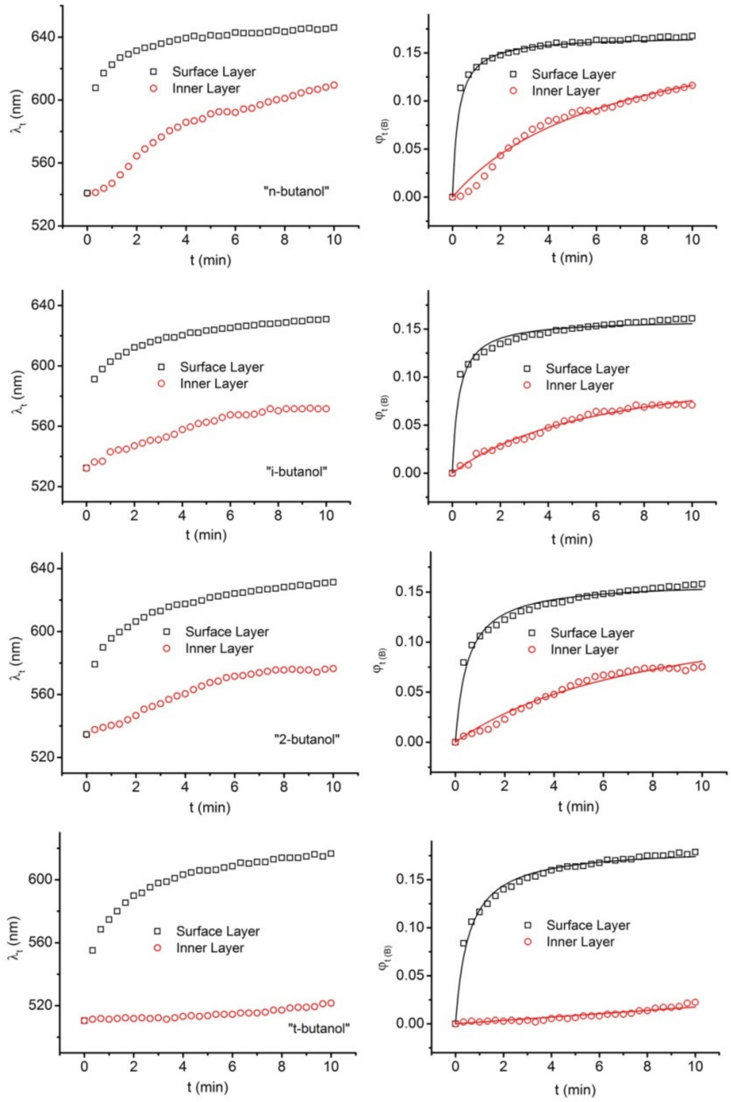

Model	MichaelisMente		
Equation	y = Vmax * x / (Km + x)		
Reduced Chi-S	1.46053E-5		
Adj. R-Square	0.98452		
		Value	Standard Err
1#	Vmax	0.1676	0.00108
1#	Km	0.2461	0.01735

Model	MichaelisMent		
Equation	y = Vmax * x / (Km + x)		
Reduced Chi-	2.75367E-5		
Adj. R-Square	0.97874		
		Value	Standard Er
2#	Vmax	0.195	0.01199
2#	Km	6.777	0.80186

Model	MichaelisMente		
Equation	y = Vmax * x / (Km + x)		
Reduced Chi-S	2.35707E-5		
Adj. R-Square	0.97385		
		Value	Standard Err
1#	Vmax	0.1600	0.00141
1#	Km	0.2812	0.02512

Model	MichaelisMente		
Equation	y = Vmax * x / (Km + x)		
Reduced Chi-S	6.29713E-6		
Adj. R-Square	0.98707		
		Value	Standard Err
2#	Vmax	0.1251	0.00551
2#	Km	6.5561	0.56422

Model	MichaelisMent		
Equation	y = Vmax * x / (Km + x)		
Reduced Chi-	2.19663E-5		
Adj. R-Square	0.97773		
		Value	Standard Er
1#	Vmax	0.160	0.00162
1#	Km	0.503	0.03687

Model	MichaelisMe		
Equation	y = Vmax * x / (Km + x)		
Reduced Ch	1.58444E-5		
Adj. R-Squar	0.97461		
		Value	Standard
2#	Vmax	0.149	0.01198
2#	Km	8.478	1.19992

Model	MichaelisMe		
Equation	y = Vmax * x / (Km + x)		
Reduced Chi	1.76501E-5		
Adj. R-Squar	0.98661		
		Value	Standard E
1#	Vmax	0.183	0.0015
1#	Km	0.553	0.03118

Model	MichaelisMe		
Equation	y = Vmax * x / (Km + x)		
Reduced Ch	4.52136E-6		
Adj. R-Squa	0.88493		
		Value	Standard
2#	Vmax	6.61202	--
2#	Km	3.78452	--

Supplementary Figure 11. Simulation of solvent diffusion in two layers from DRS patterns. “λ_t – t” and “$\varphi_{t\,(A)}$ – t” curve of four butanol isomers, and the simulation of “$\varphi_{t\,(A)}$ – t” curve using MichaelisMenten model

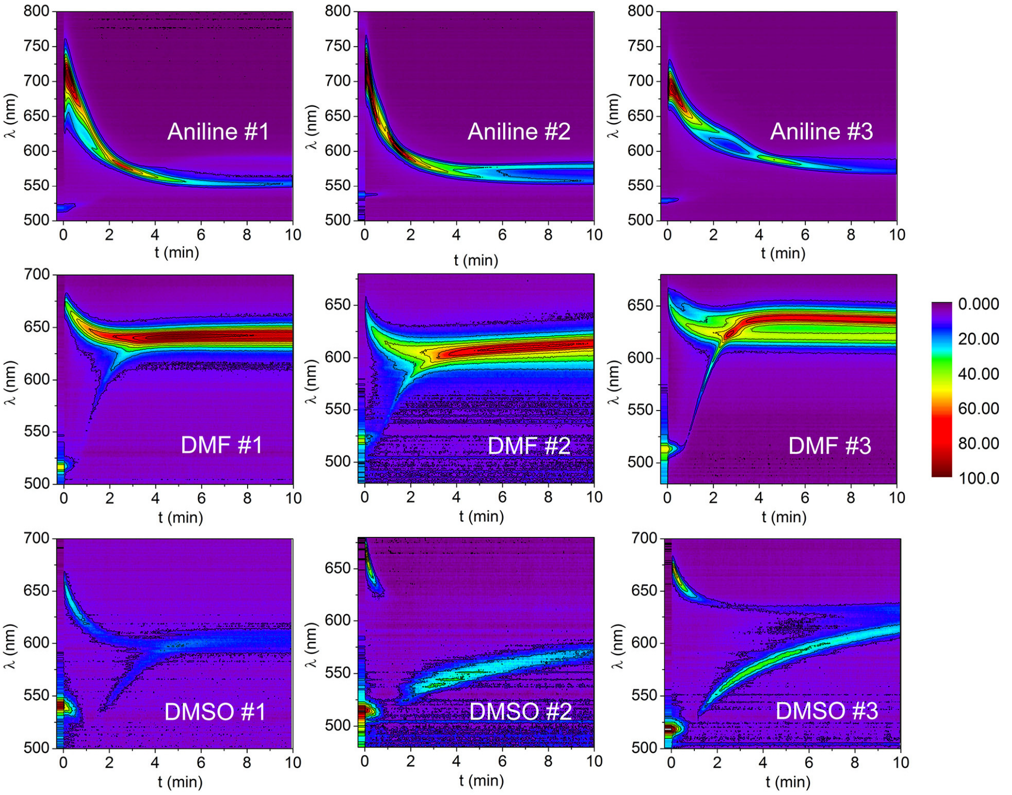

Supplementary Figure 12. DRS patterns of aniline, DMF and DMSO. Dynamic Reflection Spectra of 3 independently prepared SiO_2/PEGMA/EG photonic crystal gel responding to aniline, dimethyl formamide (DMF) and dimethylsulfoxide (DMSO).

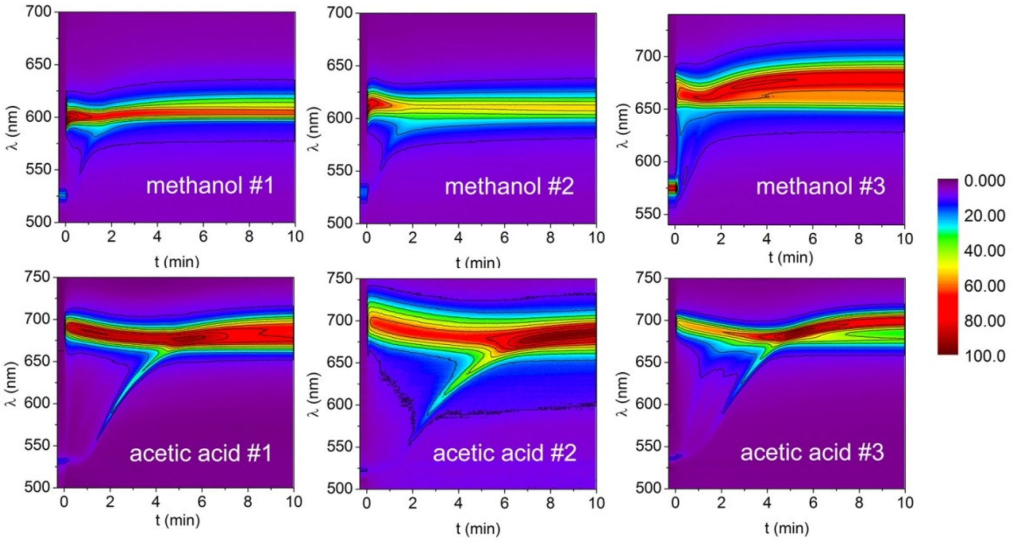

Supplementary Figure 13. DRS patterns of methanol and acetic acid. Dynamic Reflection Spectra of 3 independently prepared SiO_2/PEGMA/EG photonic crystal gel responding to methanol and acetic acid.

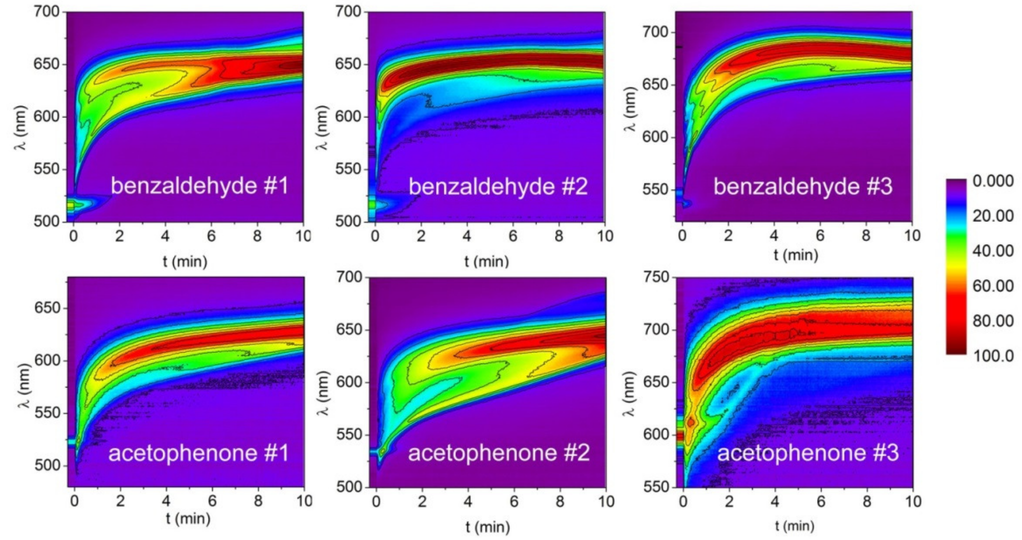

Supplementary Figure 14. DRS patterns of benzaldehyde and acetophenone. Dynamic Reflection Spectra of 3 independently prepared SiO_2/PEGMA/EG photonic crystal gel responding to benzaldehyde and acetophenone.

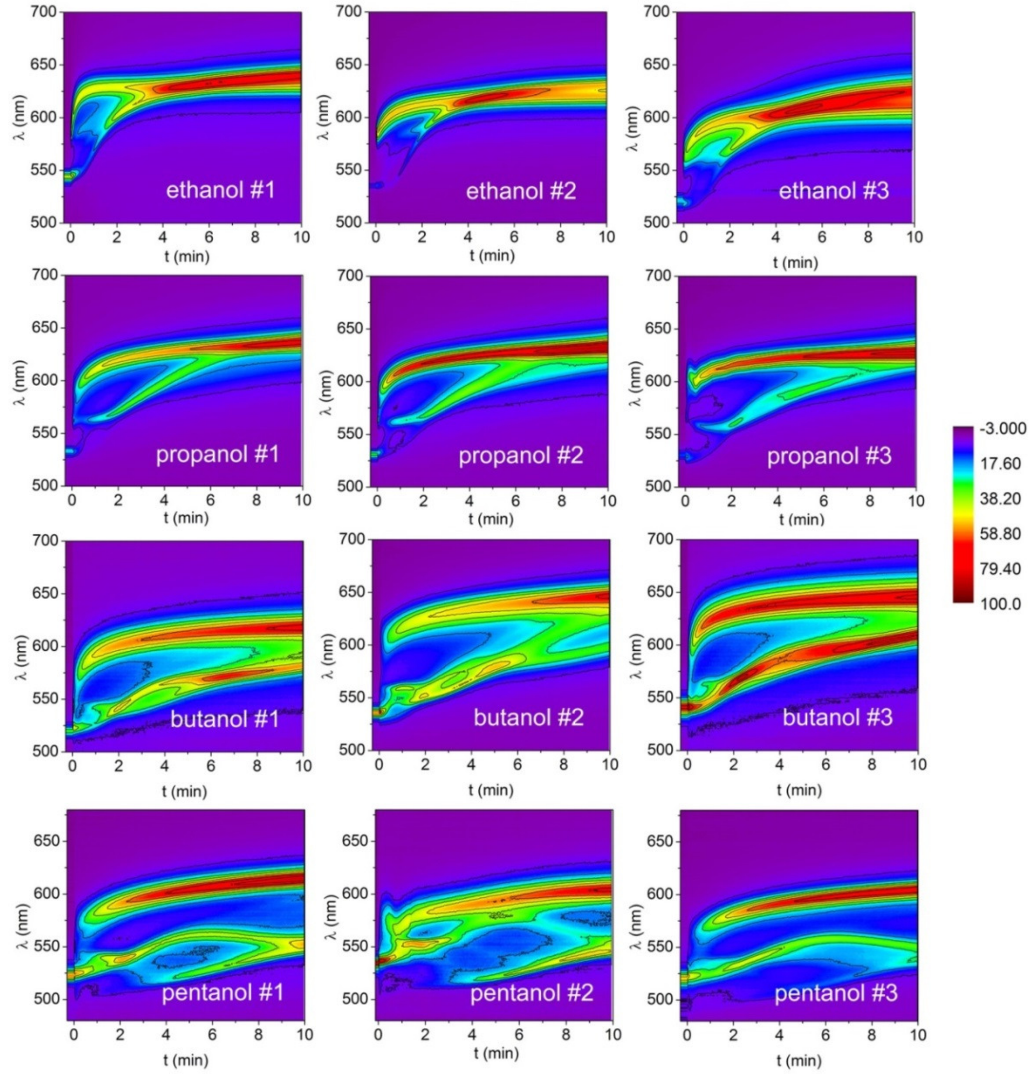

Supplementary Figure 15. DRS patterns of alcohols. Dynamic Reflection Spectra of 3 independently prepared SiO_2/PEGMA/EG photonic crystal gel responding to ethanol, propanol, butanol and pentanol.

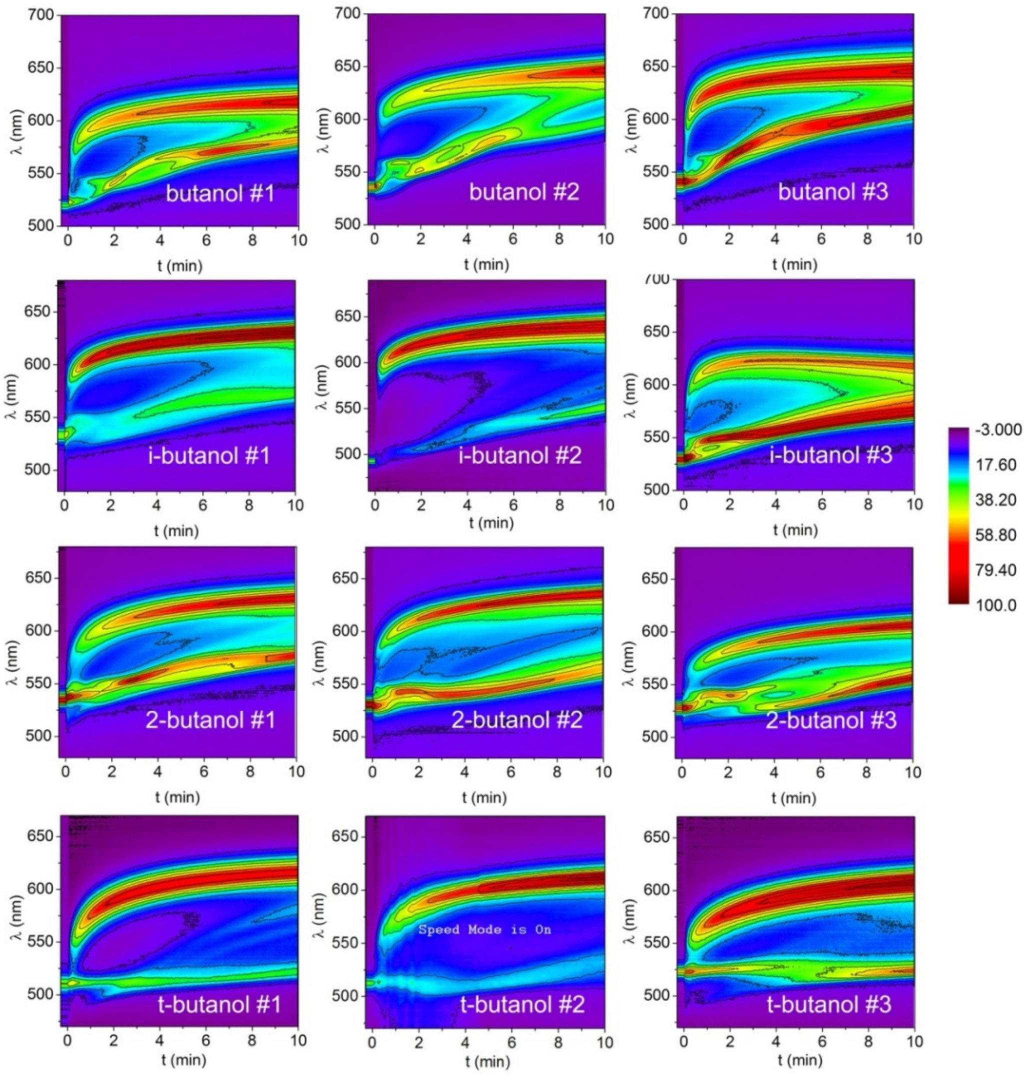

Supplementary Figure 16. DRS patterns of butanol isomers. Dynamic Reflection Spectra of 3 independently prepared SiO_2/PEGMA/EG photonic crystal gel responding to 4 isomers of butanol.

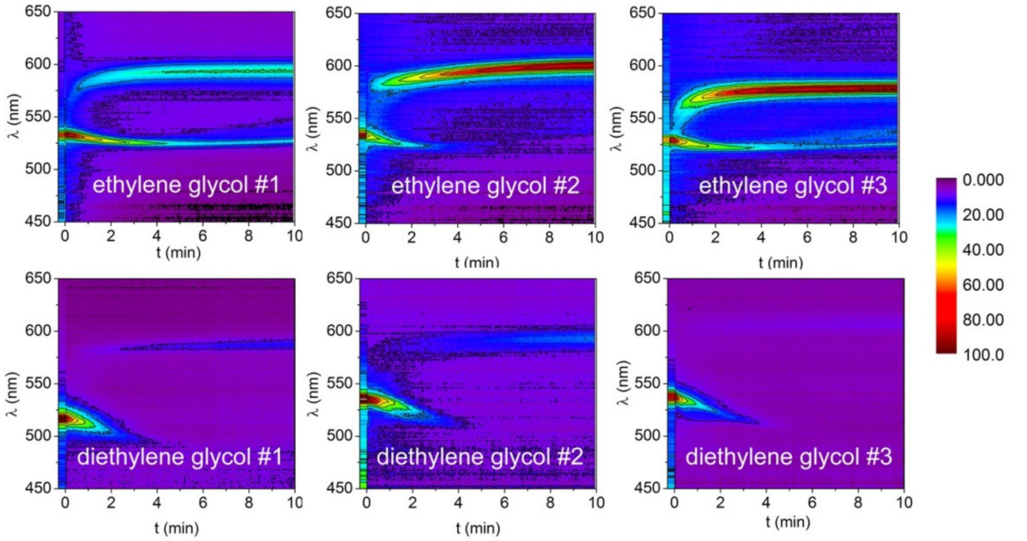

Supplementary Figure 17. DRS patterns of EG and DEG. Dynamic Reflection Spectra of 3 independently prepared SiO_2/PEGMA/EG photonic crystal gel responding to ethylene glycol (EG) and diethylene glycol (DEG).

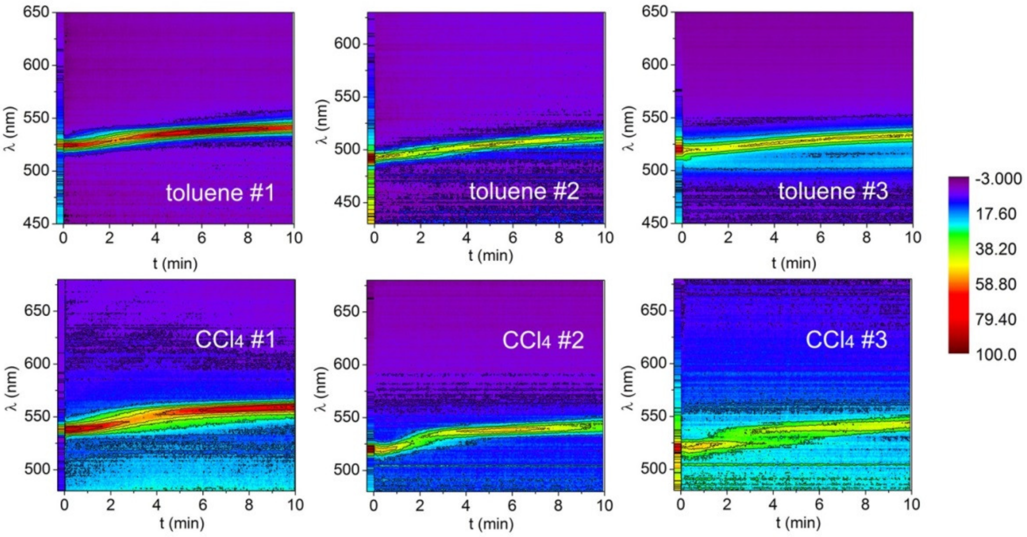

Supplementary Figure 18. DRS patterns of toluene and CCl_4. Dynamic Reflection Spectra of 3 independently prepared SiO_2/PEGMA/EG photonic crystal gel responding to toluene and carbon tetrachloride (CCl_4).

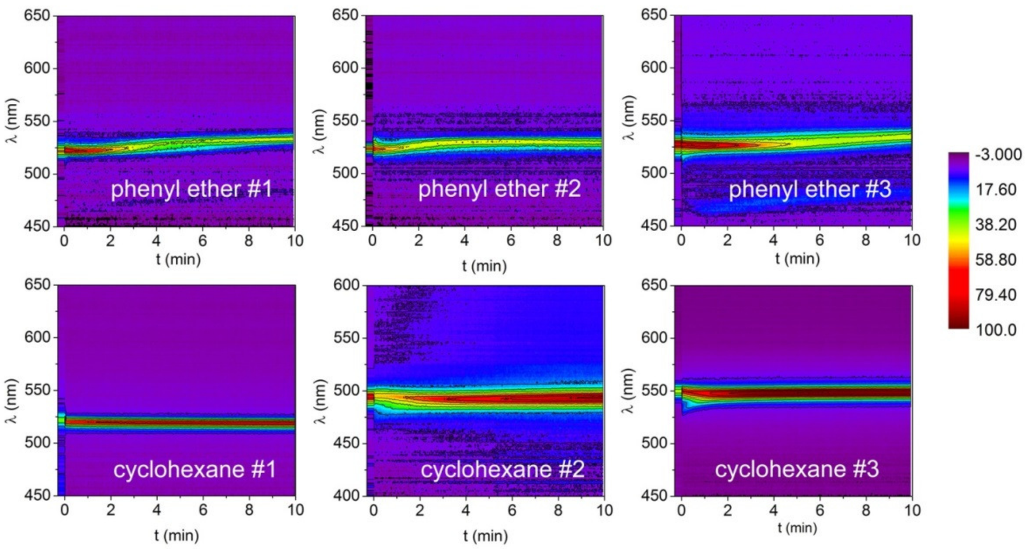

Supplementary Figure 19. DRS patterns of phenyl ether and cyclohexane. Dynamic Reflection Spectra of 3 independently prepared SiO_2/PEGMA/EG photonic crystal gel responding to phenyl ether and cyclohexane.

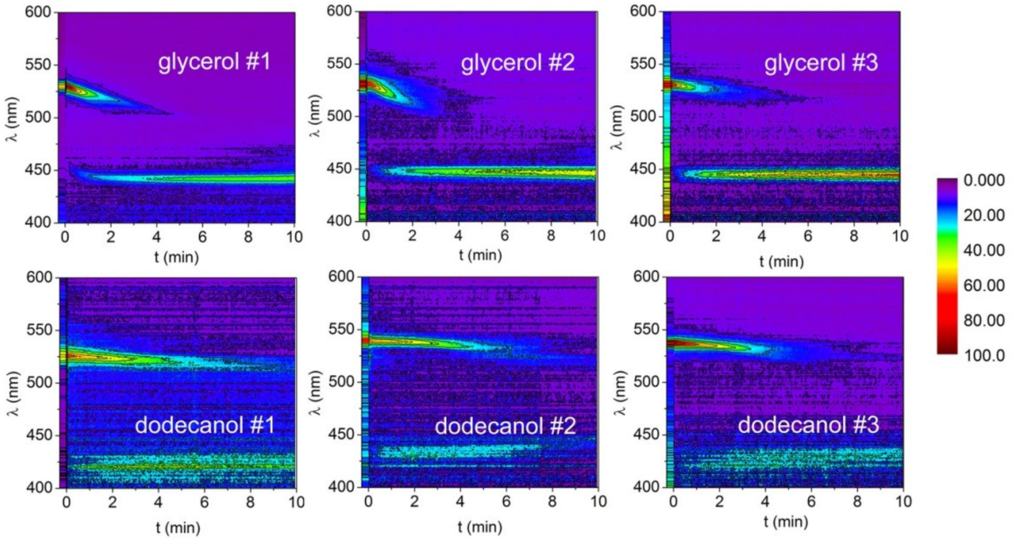

Supplementary Figure 20. DRS patterns of glycerol and dodecanol. Dynamic Reflection Spectra of 3 independently prepared SiO_2/PEGMA/EG photonic crystal gel responding to glycerol and dodecanol.

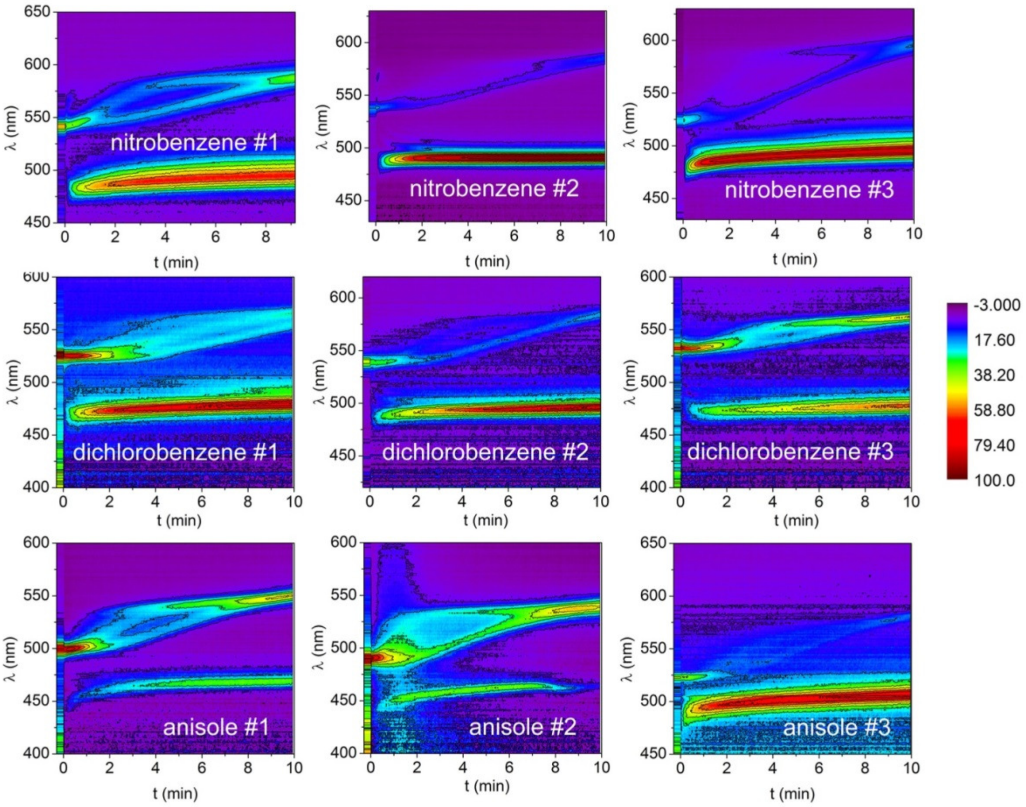

Supplementary Figure 21. DRS patterns of nitrobenzene, dichlorobenzene and anisole. Dynamic Reflection Spectra of 3 independently prepared SiO_2/PEGMA/EG photonic crystal gel responding to nitrobenzene, dichlorobenzene and anisole.

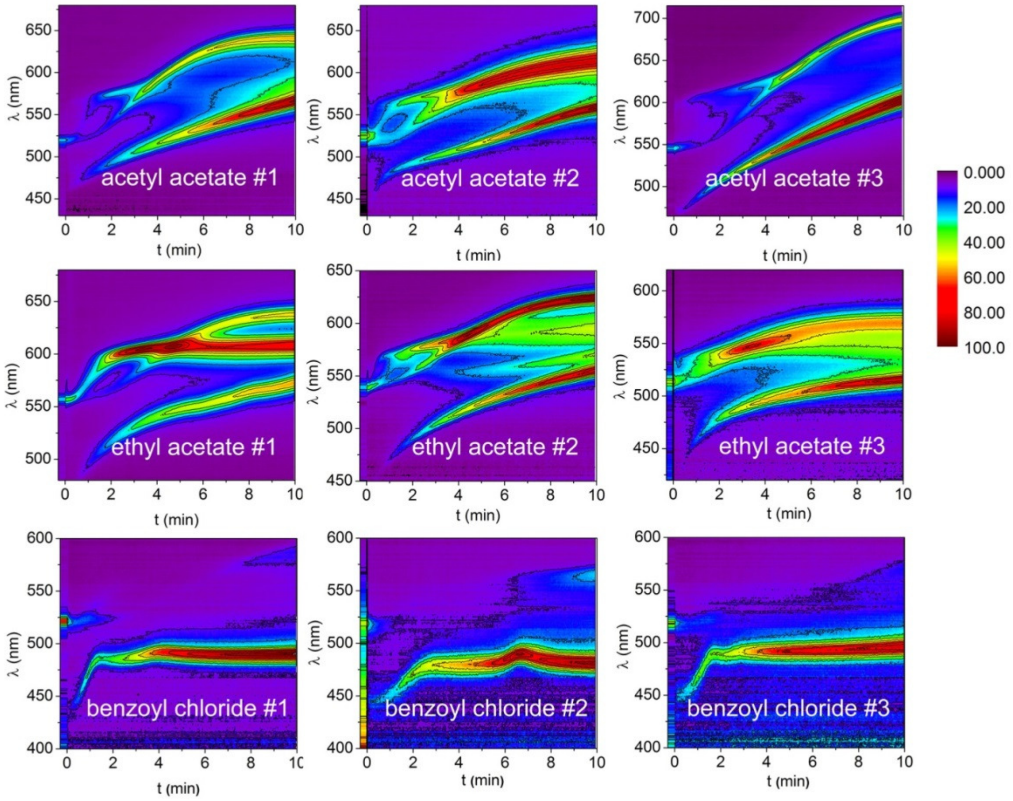

Supplementary Figure 22. DRS patterns of acetyl acetate, ethyl acetate and benzoyl chloride. Dynamic Reflection Spectra of 3 independently prepared SiO_2/PEGMA/EG photonic crystal gel responding to acetyl acetate, ethyl acetate and benzoyl chloride.

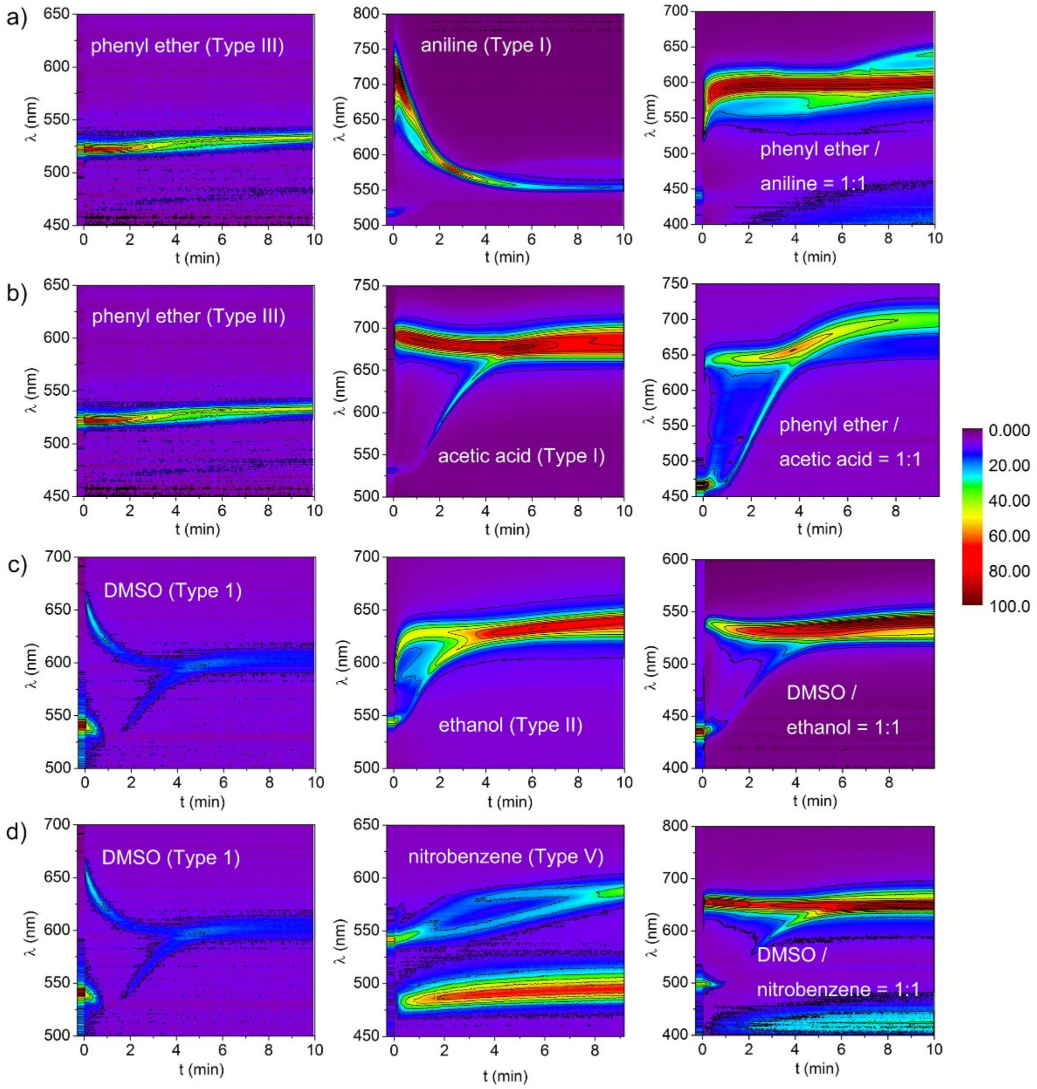

Supplementary Figure 23. DRS patterns of mixing solvents. Dynamic Reflection Spectra of SiO_2/PEGMA/EG photonic crystal gel responding to a) phenyl ether, aniline and their mixture, b) phenyl ether, acetic acid and their mixture, c) DMSO, ethanol and their mixture and d) DMSO, nitrobenzene and their mixture.

Supplementary Tables

Supplementary Table 1. Dielectric constant (ε), viscosity (η) and refractive index (n) of all organic solvents.

Solvent	ε	η / mPa·s	n	Solvent	ε	η / mPa·s	n
Cyclohexane	0.052	0.888	1.426	Methanol	31.2	0.595	1.329
Carbon tetrachloride	2.238	0.965	1.460	Ethanol	25.7	1.170	1.361
Toluene	2.24	0.587	1.497	Propanol	22.2	2.260	1.386
Phenyl ether	3.7	3.864	1.578	n-Butanol	17.1	2.950	1.399
Anisole	4.33	1.2	1.518	n-Pentanol	13.9	3.310	1.410
Aniline	7.06	1.423	1.586	iso-Butanol	17.95	4.000	1.396
Acetic acid	6.15	1.314	1.372	2-Butanol	15.5	4.210	1.397
Ethyl acetate	6.02	0.449	1.372	tert-Butanol	11.4	3.350	1.384
Acetyl acetate	20.7	0.912	1.390	Ethylene glycol	38.66	25.66	1.432
Benzoyl chloride	22.1	1.24	1.550	Diethylene glycol	31.69	35.7	1.448
1-Dodecanol	6.5	1.15	1.428	Benzaldehyde	17.9	1.53	1.545
Glycerol	42.5	1412	1.475	Acetophenone	17.39	1.642	1.534
Dichlorobenzene	6.83	1.324	1.551	Dimethyl formamide	36.71	0.802	1.428
Nitrobenzene	34.82	2.01	1.553	Dimethyl sulfoxide	48.9	1.996	1.477
Tetrahydrofuran*	7.58	0.55	1.407	Acetone*	20.7	0.316	1.359
Pyridine*	12.3	0.952	1.510	Acetonitrile*	37.5	0.375	1.344

Supplementary Table 2. The volume fraction of butanols in the surface layer (φ_1) and inner layer (φ_2), which are obtained through the simulation of "$\varphi_{t\,(A)} - t$" curve and expressed as a function of time. The diffusion speed of butanols ($d\varphi / dt$) calculated through the derivation of volume fraction (φ).

	Surface layer		Inner layer	
	φ_1	$d\varphi_1 / dt$	φ_2	$d\varphi_2 / dt$
n-butanol	$\frac{0.168t}{0.246+t}$	$\frac{1}{(1.211 + t/0.203)^2}$	$\frac{0.195t}{6.778+t}$	$\frac{1}{(5.891 + t/1.15)^2}$
i-butanol	$\frac{0.160t}{0.281+t}$	$\frac{1}{(1.322 + t/0.213)^2}$	$\frac{0.125t}{6.556+t}$	$\frac{1}{(7.238 + t/0.906)^2}$
2-butanol	$\frac{0.160t}{0.503+t}$	$\frac{1}{(1.772 + t/0.284)^2}$	$\frac{0.149t}{8.478+t}$	$\frac{1}{(7.533 + t/1.125)^2}$
t-butanol	$\frac{0.184t}{0.553+t}$	$\frac{1}{(1.736 + t/0.319)^2}$	$\frac{6.61\times 10^{12}t}{3.78\times 10^{15}+t}$	$\frac{1}{(23.94 + t/1.58\times 10^{14})^2}$

Supplementary Discussion

Simulation of "$\varphi_{t\,(A)} - t$" curve for acetophenone with single reflection band in DRS pattern

According to Bragg's Law, the instant reflection wavelength (λ_t) of the SiO_2/PEGMA/EG gel at any time during its contact with solvent can be calculated by Supplementary Equation 1 where the incident light is perpendicular to the normal direction of the gel, n_t and d_t are the instant total refractive index and instant lattice constant of the photonic gel.

$$\lambda_t = 2n_t d_t \quad (1)$$

The instant total refractive index (n_t) is usally calculated according to refractive index of each component and their corresponding volume ratios as shown in Supplementary Equation 2. When acetophenone diffuse into the gel, the instant refractive index can eventually be expressed by Supplementary Equation 3. Here, n_i (e.g. n_{EG}, n_{SiO2}, n_{PEGMA}, n_A) and n_0 are refractive index of each component and that of the original photonic gel. $\varphi_t(i)$ and $\varphi_0(i)$ are the instant volume ratio of each component and the their original value in the photonic gel before solvent infiltration. V_A and V_0 (30 μL) are the volume of infiltrated acetophenone and original gel, respectively.

$$n_t = \sum n_i\varphi_i = n_{EG}\varphi_{t(EG)} + n_{SiO_2}\varphi_{t(SiO_2)} + n_{PEGMA}\varphi_{t(PEGMA)} + n_A\varphi_{t(A)} \quad (2)$$

$$\varphi_{t(EG)} = \varphi_{0(EG)} \times \frac{V_0}{V_0+V_A};\ \varphi_{t(SiO_2)} = \varphi_{0(SiO_2)} \times \frac{V_0}{V_0+V_A};$$

$$\varphi_{t(PEGMA)} = \varphi_{0(PEGMA)} \times \frac{V_0}{V_0+V_A};\ \varphi_{t(A)} = \frac{V_A}{V_0+V_A}$$

$$n_t = \frac{V_0}{V_0+V_A}\left[n_{EG}\varphi_{0(EG)} + n_{SiO_2}\varphi_{0(SiO_2)} + n_{PEGMA}\varphi_{0(PEGMA)}\right] + n_A \times \frac{V_A}{V_0+V_A}$$
$$= \frac{V_0}{V_0+V_A}n_0 + \frac{V_A}{V_0+V_A}n_A \quad (3)$$

Since the bottom of the gel is fixed on a glass slide, the diffusion of acetophenone only causes the lattice expansion along the normal direction of the gel, so that the instant lattice constant (d_t) can be calculated by Supplementary Equation 4, where d_0 is the lattice constant of original gel.

$$d_t = \frac{V_0+V_A}{V_0}\,d_0 \quad (4)$$

With the above deduction, the instant reflection wavelength (λ_t) can finally be expressed by Supplementary Equation 5, where λ_0 is the initial reflection wavelength of the gel.

$$\lambda_t = 2\left(\frac{V_0}{V_0+V_A}n_0 + \frac{V_A}{V_0+V_A}n_A\right) \times \frac{V_0+V_A}{V_0}\,d_0 = 2n_0d_0 + 2 \times \frac{V_A}{V_0} \times n_Ad_0 = \lambda_0\left(1 + \frac{n_A}{n_0} \cdot \frac{V_A}{V_0}\right) \quad (5)$$

In the experiment of acetophenone, the refractive index of EG, SiO_2, PEGMA and acetophenone are 1.43, 1.46, 1.46 and 1.534, respectively. The initial volume ratios of EG, SiO_2 and PEGMA are 0.385, 0.40 and 0.215, respectively. The initial total refractive index (n_0) is calculated to be 1.448, and the initial reflection wavelength (λ_0) is determined to be 523.39 nm according to DRS patterns. Therefore, the volume of infiltrated acetophenone (V_A) and its real-time volume ratio are calculated by Supplementary Equation 6 and 7, respectively.

$$\lambda_t = 523.39 \times \left(1 + \frac{1.534}{1.448} \times \frac{V_A}{30}\right) = 18.4825V_A + 523.39$$

$$V_A = \frac{\lambda_t - 523.39}{18.4825} \quad (6)$$

$$\varphi_{t(A)} = \frac{V_A}{V_0 + V_A} = \frac{\lambda_t - 523.39}{\lambda_t + 31.085} \quad (7)$$

If we take the data of corresponding λ_t and t from DRS pattern of acetophenone every 20 seconds, a "λ_t – t" curve can be plotted and it can further transformed into a "V_A – t" or "$\varphi_{t\,(A)}$ – t" curve, as shown in Supplementary Figure 9. These curves can be fitted using a MichaelisMenten model, so that the volume and volume fraction of acetophenone inside the gel [$\varphi_{t\,(A)}$] at specific time can be expressed by Supplementary Equation 8 and 9, where V_{max} and K_m are two constants. Through derivation, the changing speed of acetophenone volume fraction [$d\varphi_{t\,(A)} / dt$] can be expressed by Supplementary Equation 10. It can be summarized as an empirical formula (Supplementary Equation 11), in which the constant C_1 is an important parameter showing the diffusion speed of acetophenone inside the photonic gel especially during the earlier stage of diffusion. Generally, a small value of C_1 indicates a faster diffusion and faster lattice expansion.

$$V_A = \frac{V_{max} \cdot t}{K_m + t} = \frac{6.035t}{0.843 + t} \quad (8)$$

$$\varphi_{t(A)} = \frac{V_{max} \cdot t}{K_m + t} = \frac{0.167t}{0.697 + t} \quad (9)$$

$$\frac{d\varphi_{t(A)}}{dt} = \frac{0.1164}{(0.697 + t)^2} = \frac{1}{(2.043 + {}^{t}/_{0.3412})^2} \quad (10)$$

$$\frac{d\varphi_{t(A)}}{dt} = \frac{1}{(C_1 + {}^{t}/_{C_2})^2} \quad (11)$$

Simulation of "$\varphi_{t\,(A)}$ – t" curve for butanols with two reflection bands in DRS pattern

The simulation of "$\varphi_{t\,(A)}$ – t" curve for n-butanol, i-butanol, 2-butanol and t-butanol are similar to case of acetophenone, except that the solvent diffusion and lattice expansion in the surface and inner layer, which corresponds to 2 higher reflection bands need to be considered respectively. Using the simulation of n-butanol as a typical example, one can obtain a similar expression of λ and φ_B by Supplementary Equation 12 – 15. Here, $V_{B,1}$, $V_{B,2}$, V_1, V_2 are the volume of butanol and photonic gel in the surface layer and inner layer; n_B and n_0 are the refractive index of infiltrated butanol and original gel; λ_1, λ_2 and λ_0 are the reflection wavelength of surface layer, inner layer and original gel; φ_1 and φ_2 are the instant volume ratio of butanol in surface layer and inner layer.

Through simulation and derivation, the volume fraction of n-butanol in two layers (φ_1 & φ_2) and their changing speed ($d\varphi_1 / dt$ & $d\varphi_2 / dt$) can be expressed by similar empirical formulas Supplementary Equation 16 – 19. The simulation results for all 4 butanols are summarized in Table S2. Here, the constant C_1 is an important parameter to characterize the initial diffusion speed. It shows that the diffusion and lattice expansion become faster and faster as the solvent change from t-butanol, 2-butanol, i-butanol to n-butanol, so that these four butanol isomers can be precisely distinguished according to their DRS patterns.

$$\lambda_1 = \lambda_0 \left(1 + \frac{n_B}{n_0} \cdot \frac{V_{B,1}}{V_1}\right) = 540.79 + 522.49 \times \frac{V_{B,1}}{V_1} \quad (12)$$

$$\lambda_2 = \lambda_0 \left(1 + \frac{n_B}{n_0} \cdot \frac{V_{B,2}}{V_2}\right) = 540.79 + 522.49 \times \frac{V_{B,2}}{V_2} \quad (13)$$

$$\varphi_1 = \frac{V_{B,1}}{V_1 + V_{B,1}} = \frac{\lambda_1 - 540.79}{\lambda_1 - 18.3} \quad (14)$$

$$\varphi_2 = \frac{V_{B,2}}{V_2+V_{B,2}} = \frac{\lambda_2-540.79}{\lambda_2-18.3} \quad (15)$$

$$\varphi_1 = \frac{0.168t}{0.246+t} \quad (16)$$

$$\varphi_2 = \frac{0.195t}{6.778+t} \quad (17)$$

$$\frac{d\varphi_1}{dt} = \frac{1}{(C_1+{}^{t}/_{C_2})^2} = \frac{1}{(1.211+{}^{t}/_{0.2031})^2} \quad (18)$$

$$\frac{d\varphi_2}{dt} = \frac{1}{(C_1+{}^{t}/_{C_2})^2} = \frac{1}{(5.891+{}^{t}/_{1.1504})^2} \quad (19)$$

DRS pattern of mixing solvent

It is a challenge to find the relationship in DRS patterns between the single solvent and their mixtures, since there are many combinations and possibilities which may take quite a long time to thoroughly explain the relationship. Our preliminary investigation shows that the DRS pattern of mixing solvent may retain or combine part of the geometric characteristics of their single component, which leads to a new pattern outside the five categories. Generally, we shall explain the relationship through the comparison of geometric characteristics, such as ascending/descending of reflection band ($\Delta\lambda$), color change (ΔR) and splitting/merging of reflection bands. It should be noted that at least 3 separately prepared SiO_2/PEGMA/EG photonic crystal gel are used to recognize the same solvent mixture. Most results are similar and one of them is presented in Supplementary Figure 23.

Taking the phenyl ether/aniline system as a typical example (Supplementary Figure 23a), the DRS pattern of phenyl ether is a flat reflection band extending from the original λ with little changes in color, while that of aniline is a gradually decreasing band starting from the upward jump of original λ and the band color turns red with the jumping and turns back to blue afterwards. It is interesting to find that the DRS pattern of the mixture of phenyl ether and aniline (in the ratio of 1:1) is a flat band higher than the original λ, whose color turns from blue to red and then keeps red afterwards. It is believed that the flat band with little changes in color during the 10-min test is similar to the characteristics of phenyl ether's pattern, and the upward jump of reflection wavelength and the redshift of band colors are quite similar to the characteristics of aniline's pattern.

Supplementary Methods

Materials. Tetraethylorthosilicate (TEOS, 98%), aqueous ammonia (28%), N,N-dimethylformamide (DMF, 99.5%), cyclohexane (99.5%), toluene (99.5%), acetic acid (99.5%), ethyl acetate (99.5%), acetyl acetate (98.5%), glycerol (99%), tetrahydrofuran (THF, 99%), methanol (99.5%), benzaldehyde (98.5%), benzoyl chloride (98%), dimethyl sulfoxide (DMSO, 99%), acetone (99.5%) and acetonitrile (81.0-82.0%) were purchased from Sinopharm Chemical Reagent Co. Ltd. Aniline (99.9%), carbon tetrachloride (99%), nitrobenzene (99%), pyridine (99%), propanol (99%), n-butanol (99.5%), n-pentanol (98%), iso-butanol (99%), 2-butanol (99%), tert-butanol (99.5%) and acetophenone (98%) were purchased from Aladdin Co. Ltd. Ethylene glycerol (EG, 99%), ethanol (99.9%), 1-dodecanol (99%) and anisole (99%) were purchased from J&K Co. Ltd. 1,2-Dichlorobenzene (DCB, 98.0%) was purchased from TCI Co. Ltd. Poly (ethylene glycol) methacrylate (PEGMA, Mn = 360) and diethylene glycol (DEG, 99%) were purchased from Sigma-Aldrich. Phenyl ether (99%) was purchased from ACROS. All chemicals were used directly as received without further purification.

Synthesis of SiO_2/PEGMA/EG photonic crystal gel. The photonic crystal gel for solvent sensing is prepared by fixing the metastable SiO_2 colloidal crystal arrays (CCAs) dispersed in the mixture of PEGMA monomer and ethylene glycol through photo polymerization.[1] Monodisperse SiO_2 particles are first synthesized by modified Stöber method. SiO_2 particles (81.6 mg) are well dispersed in ethanol (1 mL) through sonication and mixed with PEGMA (21.5 μL) containing photo initiator (5%) and ethanol glycol (38.5 μL). After evaporation of ethanol under 90 °C for 2 hour, the final volume fraction of SiO_2 particles, EG and PEGMA are 40%, 38.5% and 21.5% respectively. Then, the precursor (30 μL) is sandwiched between two hydrophobic glass slides with an interval of 180 μm. SiO_2 particles precipitate from the supersaturated solution and self-assemble to a metastable colloidal photonic crystal after the precursor is kept undisturbed for several minutes. Finally, the liquid precursor turns to photonic crystal gel after 20 min of UV irradiation (365 nm, 4.8 mW/cm^2).

Synthesis of SiO_2 opal solid. The as-prepared SiO_2/PEGMA/EG photonic crystal gel is first dried in vacuum to remove ethylene glycol. Then it is calcined in air at 400 °C for 2 hour and 800 °C for another 2 hours to produce a SiO_2 opal solid film. It should be noted that its size slightly shrinks compared to that of original photonic gel.

Characterizations. The dynamic reflection spectra are continuously recorded by an Ocean Optics Maya 2000 Pro spectrometer coupled to a six-around-one reflection probe with incident and reflection angles fixed at 0°. The probe faces up vertically in the holder, which is covered by a transparent glass slide. Meanwhile, the photonic crystal gel is fixed on another glass slide by UV curing of NOA61 optical adhesive, which is placed on the 1st slide, so that the gel can be sandwiched between two slides. After the reflection spectra being collected every 2 sec by the spectrometer, the tested organic solvent (200 μL) is added onto the surface of photonic gel. Here, the volume of solvent is adequate to immerse the gel considering part of the solvent will evaporate during the test. After 10 min, about 300 reflection spectra are imported to the matrix table of software "OriginPro", which is further used to plot the contour map with time (t) in x-axis, reflection wavelength (λ) in y-axis and reflection intensity (R) in color. The

microstructure of SiO_2 colloidal crystals and amorphous stacking of SiO_2 particles on the cross section of photonic gel is studied by a Phenom G2 Pro scanning electron microscope. The optical microscope images are taken by an Olympus BXFM reflection-type microscope operated in dark field mode.

Supplementary References

1. Yang DP, Ye SY, Ge JP. From Metastable Colloidal Crystalline Arrays to Fast Responsive Mechanochromic Photonic Gel: An Organic Gel for Deformation-Based Display Panels. *Adv Funct Mater* **24**, 3197-3205 (2014).
